# Microbial Characterization of Qatari Barchan Sand Dunes

**DOI:** 10.1371/journal.pone.0161836

**Published:** 2016-09-21

**Authors:** Sara Abdul Majid, Michael F. Graw, Aspassia D. Chatziefthimiou, Hanh Nguyen, Renee Richer, Michel Louge, Ali A. Sultan, Patrick Schloss, Anthony G. Hay

**Affiliations:** 1 Department of Research, Weill Cornell Medical Qatar, Qatar Foundation, Doha, Qatar; 2 Department of Microbiology, Cornell University, Ithaca, New York, United States of America; 3 Sibley School of Mechanical and Aerospace Engineering, Cornell University, Ithaca, New York, United States of America; 4 Department of Microbiology and Immunology, University of Michigan, Ann Arbor, Michigan, United States of America; University of Illinois at Chicago, UNITED STATES

## Abstract

This study represents the first characterization of sand microbiota in migrating barchan sand dunes. Bacterial communities were studied through direct counts and cultivation, as well as 16S rRNA gene and metagenomic sequence analysis to gain an understanding of microbial abundance, diversity, and potential metabolic capabilities. Direct on-grain cell counts gave an average of 5.3 ± 0.4 x 10^5^ cells g^-1^ of sand. Cultured isolates (N = 64) selected for 16S rRNA gene sequencing belonged to the phyla *Actinobacteria* (58%), *Firmicutes* (27%) and *Proteobacteria* (15%). Deep-sequencing of 16S rRNA gene amplicons from 18 dunes demonstrated a high relative abundance of *Proteobacteria*, particularly enteric bacteria, and a dune-specific-pattern of bacterial community composition that correlated with dune size. Shotgun metagenome sequences of two representative dunes were analyzed and found to have similar relative bacterial abundance, though the relative abundances of eukaryotic, viral and enterobacterial sequences were greater in sand from the dune closer to a camel-pen. Functional analysis revealed patterns similar to those observed in desert soils; however, the increased relative abundance of genes encoding sporulation and dormancy are consistent with the dune microbiome being well-adapted to the exceptionally hyper-arid Qatari desert.

## Introduction

**Fig 1:**
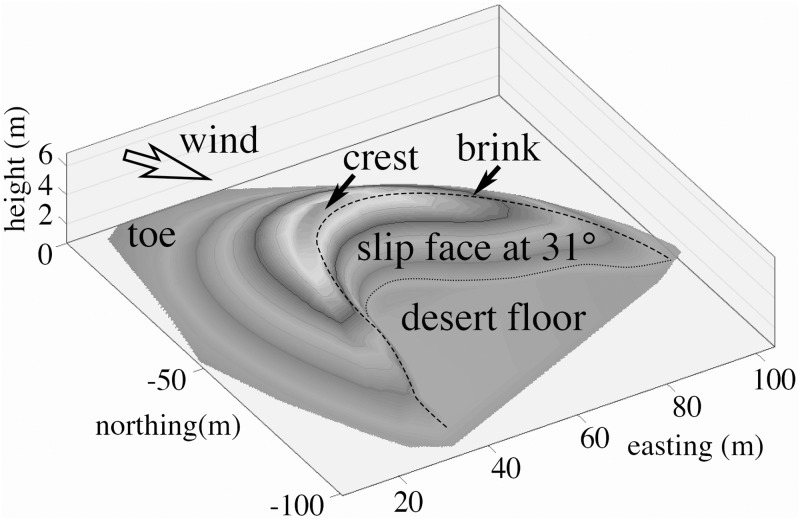
Illustration (from 2013) of Nadine dune, Lat 24.904496° Lon 51.549534.

Ten percent of the land mass between 30°S and 30°N is covered by mobile dunes [1]. Barchans are the simplest of mobile dunes. They form in desert areas where winds come predominantly from one direction. Barchan structure consists of a downwind slip face bordered by downwind-facing horns, separated from the gentler sloping windward dune face by a sharp crest [2–4], giving a familiar crescent shape when viewed from above (Fig 1). They move as a result of wind-driven sand transport over the crest and slip face of the dune, causing dune fields to migrate downwind at rates of 5–50 m annually depending on their size [3, 5, 6]. The landscape of Qatar’s southeastern region is dominated by barchans which vary in size and rate of movement [6]. The prevailing wind (Shamal) transports these dunes from the NW to the SE, scouring the landscape as they pass. The blowing sands can bury buildings and transportation infrastructure, clog sea lanes, and may contain cyanobacterial neurotoxins that have the potential to impact human health [7–9]. Dunes may also contribute to aeolian dust that has a negative impact on respiratory and cardiopulmonary health [10,11]. We were therefore interested in the nature of the microbial community in Qatari sand dunes.

The microbiology of desert soils has been increasingly studied [12–22], though dunes in general and barchans in particular have received less attention. Stationary dunes may have distinct biota from mobile dunes as they are subject to different forces. Gommeaux et al. [23] estimated a population of 2.2 ± 0.2 x 10^5^ bacterial cells g^-1^ in sand from stationary dunes in Morocco, while Yu et al. [24] estimated a population of 1.8 to 43.9 CFU x 10^3^ g^-1^ in Israeli dunes. Gommeaux et al. [23] used 16S rRNA gene sequence analysis of 34 isolates to assess culturable bacterial diversity in Moroccan dune sands, revealing that the most abundant phyla were *Actinobacteria*, *Firmicutes*, *Proteobacteria* and *Bacteroidetes*. Cloning and sequencing of 16S rRNA genes amplified from sand genomic DNA revealed similar results. More recently, others [13, 25, 26, 27, 28] have performed sequencing of 16S rRNA gene amplicons from dunes in the Gurbantunggut, Namib, Gobi and Taklamakan deserts. In addition to *Proteobacteria*, *Firmicutes*, *Actinobacteria* and *Bacteroidetes*, *Cyanobacteria* were also abundant in the Gurbantunggut sands due to the presence of Biological Soil Crusts (BSCs) [13, 26].

Barchan dunes, however, have received little attention [29]. We recently observed that much of the interior of the barchan dune we studied in Qatar remained moist and cool enough to support microbial activity despite inhospitable ambient conditions at the surface of the dune [30]. Given that dune moisture content varies with depth [30], and that dunes migrate at different rates depending on their size, we sought to understand how these factors affect microbial communities on the dunes.

This study represents the first detailed characterization of sand microbiota in migrating barchan dunes. Our questions were primarily: 1) What is the bacterial density within a barchan? 2) Does the microbial community change with depth? 3) Are there differences in community composition based on location along the dune? and 4) What factors influence community structure? Bacterial communities were studied through direct counts and cultivation, as well as by deep 16S rRNA gene amplicon and metagenomic sequence analyses in order to address these questions.

## Materials and Methods

Detailed direct microscopic observations, culturing and sequencing of isolate 16S rRNA genes were performed on samples taken from a single dune in June of 2013. This dune, which we named Nadine (see S1 Table for GPS coordinates), was chosen because it had been extensively studied with respect to sand temperature and humidity, and had yielded clear evidence of *in situ* microbial activity [30]. Shotgun metagenomic sequence analysis was performed on DNA extracted from Nadine sands and on DNA extracts from the sands of its nearest neighbor, a smaller dune located approximately 1 km to the north, which we named Michel (S1 Fig and S1 Table). Additionally, deep sequencing was performed on the 16S rRNA gene amplicons from 18 dunes including Nadine and Michel, collected between May of 2011 and June of 2013 (S1 Fig and S1 Table). Organic matter content and chemical composition was also obtained for 16 of these dunes (see S2 Table).

### Field Location and Sampling

No permissions were required to conduct this study, and no endangered or protected species were involved. The Ministry of Environment in Qatar requires permission for fieldwork in protected areas. The area we surveyed is not protected. The 18 dunes were selected based on their varying sizes and distance from our main site where we had been studying temperature and moisture dynamics in a single dune [30]. An intensive sampling effort was carried out for 9 dunes. Samples from these 9 dunes were aseptically collected from 3 locations on the windward face at the crest, in the middle (mid) and at the base of the dune in 5 cm depth increments from 0–20 cm. In addition, a single floor sample was taken adjacent to each dune, for a total of 13 samples per dune. To increase dune coverage, an additional 9 dunes were sampled once from the middle of the dune at a depth of 10–15 cm. Although surface temperatures were not recorded at the time of collection, they track with ambient temperature on a diurnal cycle. The temperature of sand 10-15cm below the surface remains at a nearly constant temperature of 22°C year round. Samples were immediately placed in sterile plastic disposable bags (approximately 750 g) for moisture analysis or sterile conical tubes (50 g) for microbial characterization and kept on ice in the field and during transport to the laboratory (~ 2 hours). Once in the laboratory, samples were weighed and stored at 4°C prior to cultivation, moisture content and pH analyses, for a maximum of 10 days, or stored immediately at -20°C for DNA extraction.

### Moisture Content

Sand (approximately 300–500 g) was poured into foil trays and dried at 105°C for 24 hours for moisture content analyses. Foil trays were allowed to cool in a desiccator then weighed. Moisture content was then calculated as the percent difference of wet to dry sand weight. In preliminary experiments we performed moisture content analysis in triplicate but found negligible variability (< 1% standard error of the mean), thus, thereafter, we only determined the moisture content of a single sample per depth for the base, middle and crest locations of a dune (13 samples).

### pH and Chemical Composition

The pH of sand extracts (4 g of sand in 8 ml dIH_2_O) from 16 dunes was determined using a ThermoOrion pH meter model 410 (Cole Parmer, USA). The organic matter content and chemical composition analyses (total nitrogen, ammonia, nitrate, arsenic, boron, barium, beryllium, calcium, cadmium, cobalt, chromium, copper, iron, potassium, lithium, magnesium, manganese, molybdenum, sodium, nickel, phosphorus, lead, sulfur, selenium, titanium, vanadium, and zinc) were performed at the Cornell Nutrient Analysis Laboratory (Ithaca, USA). Organic matter content was determined using the loss on ignition method [31]. Chemical composition was determined via ICP-EOS of sand that had undergone microwave-assisted aqua regia digestion. Although fine soil structure characterization plays an important role in structuring edaphic communities, it was impossible to measure it for the Qatari dune sands because they were completely unstructured.

### Direct Counts

Direct cell counts were performed on samples from Nadine and determined using 2 different methods: 1) direct microscopic observation of individual grains mounted on a slide and 2) direct microscopic observation of cells from a sand extract retained on an Isopore polycarbonate membrane filter (0.2 μm, 25 mm, Millipore, UK). The on-grain method was chosen because preliminary results revealed that many microbes remained attached to grains even after vortexing. However, since the majority of published soil analyses involve enumeration of soil extracts, we still performed both extract and direct-on-grain enumeration to be able to make comparisons. The fluorescent stain was prepared with a final concentration of 50 μM Propidium Iodide (Molecular Probes, USA) and 5 μM SYTO 9 green (Life Technologies, USA).

For direct microscopic observation of individual grains, slides were warmed on a hot plate at 55°C, sprinkled with 50–100 grains of sand per sample and covered with a mixture containing 75 μl of 1% Bacto Agar (BD, France) and 25 μl Propidium Iodide/SYTO 9 fluorescent stains, then allowed to cool at room temperature. For each sample, twenty grains (replicates) were examined for the presence of fluorescent green cells using a Zeiss Colibri Axio LSM 710 microscope (Zeiss, Germany) at a total magnification of 200X and excitation and emission wavelengths of 470 nm and 530 nm respectively. With this protocol, only the upper half of grains were visible. The number of cells on the lower half of the grain was assumed to be the same as on the upper half [23], thus the number of cells observed was multiplied by 2. An estimate of the direct counts per gram was obtained by multiplying the average number of cells per grain by the average number of sand grains per gram. We assumed that sand grains were spheres of material density rhos and diameter d. We neglected the mass of air, water and microbes, and calculated the number per unit mass as 6 (**π** d^3^ rhos)^-1^. For Qatari sands, rhos = 2630 kg (m^3^)^-1^, and d = 350 μm on the windward slope [30]. Hence we calculated approximately 17,000 grains per gram of sand.

For microscopic observation of cells from sand extracts, 4 g of sand from Nadine were mixed with 8 ml of 0.9% sterile NaCl in 15 ml HDPE conical vials. The vials were vortexed for 1 min and left to settle on ice for 30 min. Subsequently, 1 ml of this sand extract was pipetted onto a filter that had been mounted on an Epifluorescence Stainless Steel Filter Holder (Millipore, UK) attached to a Pyrex 500 ml flask with a vacuum pump. After filtration, the filter was mounted on a slide, covered with 10 μL of the Propidium Iodide/SYTO 9 mixture and a cover slip, then incubated in the dark for 5 min. For each sample, twenty fields were examined at a total magnification of 400X. We also attempted to analyze ATP in sand slurries and extracts using FL-AA ATP Bioluminescent Assay Kit (Sigma-Aldrich, USA); however, we found ATP to be at or below the kit’s detection limit (2 x 10^−12^ moles/liter, data not shown).

One-Way ANOVA was performed using SPSS [32]. The Post Hoc Test using the Bonferroni model was performed to determine if there were differences between sample locations (i.e. difference between base, mid, crest) and between sample depth.

### Culturing of Sand Bacteria

Culturing of sand bacteria was performed on samples from Nadine. Individual grains were picked with a sterilized dissecting needle that had been dipped in sterile 30% Glycerol (Sigma-Aldrich, USA) which facilitated grain attachment, and then plated on 0.1X R2A Agar (BD, France) and Czapek Dox Agar (HiMedia, India). Plates were incubated in the dark at room temperature (21°C) and colonies on individual grains (n = 504) were counted every four days for a total of sixteen days. Other media such as Potato Dextrose, Rose Bengal, and SNAX were initially tested, however, 0.1X R2A and CDA yielded the largest number of colonies and were used exclusively thereafter. For filter analyses, 4 g of sand were vortexed in 8 ml of 0.9% sterile NaCl, and 2 ml of extract were placed in a sterile reagent reservoir (VWR, USA). Serial 1:10 dilutions were prepared in 96 well plates, then 5 μL of each dilution were deposited onto 241 x 241 x 20 mm bioassay dishes (VWR, USA) containing solid Czapek Dox (CDA) or 0.1X R2A media using a 12 channel pipette (each sample was plated 12 times). Plates were stored in the dark at room temperature (21°C) and Colony Forming Units (CFUs) were counted every four days for a total of sixteen days. One-Way ANOVA was performed as above to determine significant differences between CFUs by sample depth and location.

### 16S rRNA Gene Amplicon Sequence

Isolated colonies were randomly selected from both single grain cultivation and serial dilution plates and sequenced after PCR amplification using the primer set 8F (5’- AGA GTT TGA TCC TGG CTCAG -3’) [33] and 338R (5’-GCT GCC TCC GGT AGG AGT -3’) [34]. These sequences gave resolution to the genus level. PCR products were sequenced using a Sanger Sequencer at the Institute of Biotechnology at Cornell University (Ithaca, NY). Isolates were identified based on BLAST [35] sequence similarity to organisms within the NCBI’s reference 16S rRNA gene sequence database.

MO BIO PowerSoil^®^ Soil DNA Isolation Kit (MO BIO, USA) was used to extract DNA from 0.25 g of sand for 16S rRNA gene amplification and sequencing. PCR was performed using barcoded primers following the strategy of Kozich *et al*. [36], except that we targeted the V6 region of the gene using forward primer 5’- ACA CTC TTT CCC TAC ACG ACG CTC TTC CGA TCT (barcode) ACT YAA AKG AAT TGA CGGG -3’ and reverse primer 5’- CGG TCT CGG CAT TCC TGC TGA ACC GCT CTT CCG ATCT (barcode) ACR ACA CGA GCT GAC GAC -3’ (see S1 Table for barcodes). PCR was performed using an Applied Biosystems 2720 Thermal Cycler (Thermo Fisher Scientific, USA) using the following protocol: 2 min at 98°C, followed by 20 cycles of 20 s at 98°C, 15 s at 50°C, and 5 min at 72°C. PCR products were gel purified using PureLink^®^ Quick Gel Extraction Kit (Invitrogen, USA) to eliminate primer-dimers. These PCR products were then re-amplified with adapters in preparation for Illumina sequencing using forward adapter (5’- AAT GAT ACG GCG ACC ACC GAG ATC TAC ACT CTT TCC CTA CACGA-3’) and reverse adapter (5’-CAA GCA GAA GAC GGC ATA CGA GAT CGG TCT CGG CAT TCC TGC TGAAC -3’) at 10 μM [36]. The PCR products were checked for amplicon size by agarose gel electrophoresis, pooled based on band intensity, concentrated and then gel purified. After library construction, the sample was spiked with 50% PhiX (Illumina, USA) and sequenced on an Illumina MiSeq instrument, implementing V2 chemistry, with paired-end 250 nt reads according to the manufacturer's protocol at Cornell’s Institute of Biotechnology.

16S rRNA gene sequence reads were processed using the MOTHUR software package (version 1.33.3; [37]) as described previously [36, 38]. Reads were aligned and joined based on quality scores and trimmed to 275 bp. Reads were then aligned to a SILVA-based 16S rRNA gene reference database [39] and passed through UChime [40] to remove chimeric sequences. Operational Taxonomic Units (OTUs) were annotated to the taxonomic level of genus using the Ribosomal Database Project (RDP, release 9) sequence classifier [41]. The classification process used 100 bootstrap iterations and an 80% confidence cutoff [39]. A small number of samples returned an anomalously low number of sequences and were excluded from further analysis, resulting in varying numbers of samples from each dune (see S1 Table).

### Shotgun Metagenomic Sequencing and Analysis

In order to obtain DNA from a larger sample of sand that would be more representative than the smaller samples used for deep 16S rRNA gene amplicons, the MO BIO PowerMax^®^ Soil DNA Extraction Kit (MO BIO, USA) was used to extract DNA from 20 g of sand from 2 dunes (2 x 10 g extractions per dune) for metagenomic sequencing. Due to limited resources, only 2 of the 18 dunes were selected for metagenome analysis. DNA was concentrated by performing Isopropanol Precipitation [42]. Library construction was performed using the Nextera XT kit from Illumina. After library construction, the sample was spiked with 50% PhiX and sequenced on an Illumina MiSeq instrument using paired 150 nt reads according to the manufacturer's protocol at the sequencing facility of Weill Cornell Medical in Qatar. The dune Nadine was chosen for metagenomic analyses since we had been studying its physical characteristics [30]. For comparison, we also sampled a nearby-dune (Michel) that showed more evidence of human and domestic animal disturbance.

Metagenome sequences were uploaded directly to the software environment MG-RAST [43]. Initially, re-duplication and low quality reads were removed by applying the quality control (QC) filter to the raw sequence data amalgamated with their associated quality scores (FASTQ format) [43].

The taxonomic composition of Michel and Nadine were compared on the basis of 16S gene amplicon and metagenomics sequences. For the metagenomes, taxonomic composition was identified using both Phyloshop [44] which identifies 16S rRNA gene reads in the metagenomes and determines taxonomic composition from these reads only, and MG-RAST, which determines taxonomic composition based on the phylogeny of functional gene reads. Metagenome sequences were classified in MG-RAST using Representative Hit Classification against the M5NR protein database, using a maximum e value of 1x10^-5^.

Analysis of the functional composition of the metagenomes was determined using hierarchical classification, with data being compared to MG-RAST's Subsystems. Additional metagenomes from Fierer *et al*. [15] were compared to our metagenomes and visualized using the software STAMP [45] to identify differences in functional pathway abundances and to more closely examine taxonomic differences in desert sands and soils.

### Community Analysis

All statistical analyses were carried out using the vegan package for R as well as the base features of R [46, 47] unless otherwise noted. The 16S rRNA gene samples were rarefied to 15,710 sequences each prior to calculating the Shannon diversity index and Bray-Curtis dissimilarity metric [48]. Factorial or nested ANOVA tests were used where appropriate to test whether the Shannon diversity index of the communities varied across and within individual dunes. Permutational multivariate analysis of variance (PERMANOVA) was used to determine whether bacterial community compositions varied significantly across dunes, within individual dunes, or according to dune size; significance was determined by 1,000 permutations of the community data. This statistical test has been found to be robust to heterogeneities in community dispersion [49]. A Mantel test using Spearman correlation with 1,000 permutations was used to assess whether community dissimilarity between pairs of dunes (using the average dissimilarity between all samples from a given dune pair) was correlated with the pairwise distances between dunes. Pairwise dissimilarities between microbial communities were visualized using non-metric multidimensional scaling (NMDS) and Pearson correlation was used to identify specific taxa contributing significantly to the observed ordination results. Similarity profile analysis [50] was conducted using the Fathom toolbox for Matlab [51] with average-linkage clustering and a cluster significance threshold of 0.001 based on 1,000 permutations in order to identify statistically significant clusters of communities. Group centroids and associated 95% confidence intervals were calculated in NMDS space for clusters identified by similarity profile analysis using vegan. Linear discriminant analysis (LDA) was conducted using LEfSe to determine whether the relative abundance of OTUs varied significantly across and within the dunes [52].

Measurements of organic matter, nitrogen species, moisture content, pH, and metals were obtained for a subset of 36 samples from 16 dunes (see S2 Table for a full list of measured abiotic parameters). These samples were chosen such that abiotic parameters were measured for the range of dune sizes, locations, and depths. In addition, we defined *a posteriori* a relative *Gammaproteobacteria* abundance as a univariate variable using the multivariate community composition dataset. Relative *Gammaproteobacteria* abundance was defined as the proportion of sequences within each sample that were classified as *Gammaproteobacteria* at the taxonomic level of class. A *t*-test was then conducted in Excel to compare the percent *Gammaproteobacteria* in dunes down wind of camel farms and those that were not. The dunes were categorically defined as downwind (within 3 km) or not by visual inspection of S1 Fig. ANOVA and multiple linear regression were used to model alpha diversity as a function of these variables. Distance-based linear modeling (distLM) [53] using Akaike's corrected information criterion as well as Pearson correlation to the NMDS ordination were used to determine which of these variables correlated with observed beta diversity patterns.

## Results

### Direct Counts

Table 1 shows cell counts per gram of sand from two different depths (0–5 cm and 15–20 cm) at the base, middle and crest of Nadine, as well as from the adjacent desert floor. On average, each grain of sand from Nadine had approximately 30 live cells (S2A Fig), and each gram of sand had an average of 5.3 ± 0.37 x 10^5^ cells, though there were significantly more cells on grains from the base of the dune where moisture content was higher (Table 1, (P<0.05, One Way ANOVA)). While there was a significant difference in the number of cells per gram of sand from the base when compared to the middle and crest, there was no significant difference in the number of cells per gram as a function of sample depth.

**Table 1:** Average on-grain cell counts per gram of sand (± Standard Error) retrieved from floor, base, mid and crest of Nadine (column 2). Column 3 shows the percentage of colony forming grains (of 504 grains tested) for various dune locations, as well as estimated colony forming grains per gram of sand. Column 4 shows the number of CFUs in sand extracts. Column 5 shows the moisture content per gram of sand.

	On-Grain Counts		On-Grain CFU		Extracts CFU	Moisture
Sample location & depth	No. cells grain^-1^	No. cells gram^-1^ ± error (10^5^)	Colony forming grains (%)	Colonies gram^-1^ (x10^3^)	CFU gram^-1^ (x10^3^)	Moisture content (%) gram^-1^
Nadine Floor	27	4.52 ± 0.68	21	3.57	0.48	0.19
Nadine Base 0–5 cm	40	6.82 ± 0.69	71	12.1	4.29	0.37
Nadine Base 15–20 cm	39	6.68 ± 0.82	57	9.69	2.42	0.32
Nadine Mid 0–5 cm	27	4.64 ± 0.57	31	5.27	2.97	0.29
Nadine Mid 15–20 cm	29	5.00 ± 0.58	40	6.80	2.11	0.42
Nadine Crest 0–5 cm	25	4.17 ± 0.58	35	5.95	1.06	0.28
Nadine Crest 15–20 cm	29	4.98 ± 0.61	28	4.76	0.60	0.20

The average number of live cells detected on individual sand grains was 10 times higher than the average of 5.3 ±0.7 x 10^4^ live cells g^-1^ of sand we observed on filters retaining sand extracts (see S3 Table). This was likely due to a combination of cell lysis during vortexing and incomplete removal of bacteria from grains. Staining revealed that nearly 30% of the cells detected before vortexing were still attached to the grains after vortexing (data not shown).

### Culturable Microbes

On average, 65% of the grains screened from the base of Nadine (0–20 cm), yielded colonies on 0.1X R2A or CDA plates within 16 days of incubation (Table 1, S2B Fig). Only 40% of grains, however, from all locations and depths on Nadine yielded colonies. Based on the conservative assumption that each grain-derived colony arose from a single culturable bacterium and that 40% of grains yielded a colony, there would be 6.8 ± 1.0 x 10^3^ CFU g^-1^ of sand (0.4 x 17000 grains gram^-1^) in Nadine. Consistent with the direct counts, CFUs were highest in sand samples obtained from the base of Nadine (Table 1). Approximately 10% of the cultured grains yielded raised, filamentous colonies that were indicative of *Streptomycetes*. Of the cells visualized directly on grains, 1.2% were culturable when the grains were plated directly.

Plating of serial dilutions of sand extracts revealed that there was an average of 2.0 ± 0.5 x 10^3^ CFU g^-1^ of sand (Table 1). Though the absolute numbers were approximately one third of those obtained with the single grain cultivation method (range 2-8x), the same trend was apparent, with the base yielding the highest number of culturable cells. Cell culturablity from the extracts was 3.8% when normalized to filter direct counts, but only 0.4% when normalized to grain counts. This suggests that the dislodged cells were nearly ten times more culturable than those cells that remained attached to the grains (S3 Table).

### 16S rRNA Gene Sequence Analysis from Isolates

A total of 64 isolates from single grain cultivation and plated serial dilutions were randomly selected for 16S rRNA gene sequencing. Analysis revealed that the 16S rRNA gene sequences of most of these stains were at least 97% similar to representatives of more than 31 different genera from three main phyla (S4 Table for name and accession number of closest relatives). Approximately 15% of isolates were *Proteobacteria*, mainly from the families *Pseudomonadaceae*, *Rhodospirillaceae*, and *Erythrobacteraceae*. The majority of isolates, however, were *Actinobacteria* (58%) and *Firmicutes* (27%) with the most abundant families being *Micrococcaceae*, *Streptomycetaceae*, and *Bacillaceae*. Two of the isolates were only distantly related (92% similarity) to characterized genera of *Actinobacteria*.

### 16S rRNA Gene Amplicon-sequencing

**Fig 2:**
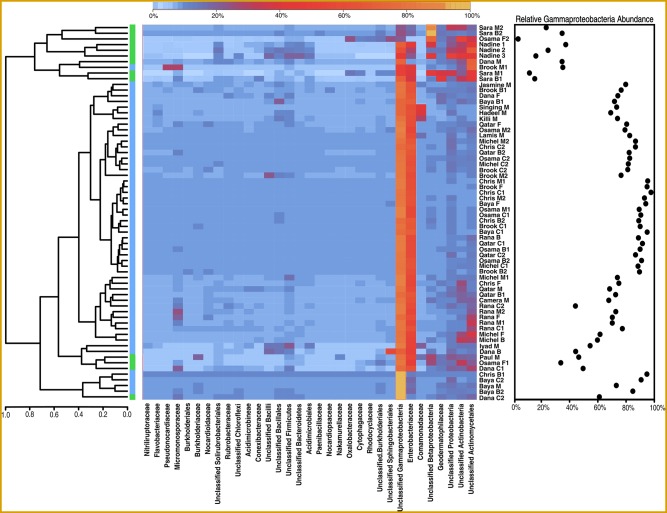
Heatmap showing the most abundant OTUs in all samples. OTU names are given at the lowest available taxonomic level; OTUs that could not be classified below the level of order are termed Unclassified. Dendrograms were generated using hierarchical clustering with complete linkage. Plot indicates the relative fraction of sequences in each sample that were classified as *Gammaproteobacteria* at the level of class. Sample key: dune name; location on dune face (C = crest, M = middle, B = base); sample number.

High-throughput sequencing of the 16S rRNA genes amplified from sand DNA yielded >3.75 million paired-end reads (250bp). Following sequence processing using MOTHUR (version 1.33.3; [37]) and rarefaction, ~990,000 sequences representing 235 OTUs annotated to the level of genus were characterized (S3 Fig, S1 Table). These sequences were derived from 66 bacterial communities from 18 dunes. The bacterial communities of all dunes consisted predominantly of *Proteobacteria* (83% of sequences classified at the phylum level) (Fig 2). *Actinobacteria* were the next most abundant community members (13%), with the remainder consisting primarily of *Firmicutes*, *Bacteroidetes*, and *Chloroflexi*. In all but three samples, *Gammaproteobacteria* made up >25% of the total bacterial community; more than half of the samples consisted of >75% *Gammaproteobacteria*. Although a large portion of these sequences were unclassified beyond the level of class, 40% of *Gammaproteobacteria* sequences were from the family *Enterobacteriaceae*. The *Actinobacteria* were dominated by members of the order *Actinomycetales*, of which numerous families were present.

**Fig 3:**
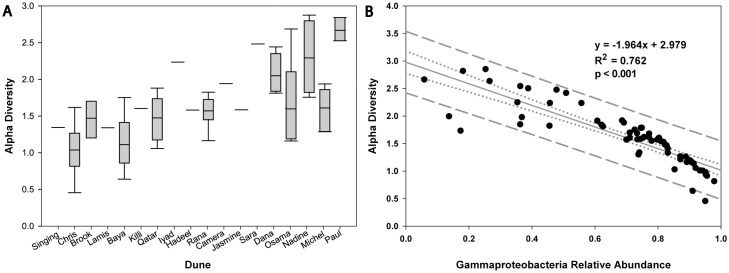
**A**. Alpha diversity, as measured by the Shannon diversity index at genus-level resolution. Samples were grouped according to their dune of origin. **B**. Linear regression of alpha diversity by the proportion of sequences classified as *Gammaproteobacteria* out of the total number of sequences in each sample.

Alpha diversity, measured using the Shannon diversity index at genus-level resolution, varied significantly between dunes (p = 0.0004; Fig 3 and Table 2) and according to dune size (measured as horn-to-horn distance; r^2^ = 0.17, p = 0.0011), but was not significantly different between different locations on the same dune regardless of where on that dune the community was sampled. Alpha diversity was only weakly correlated with pH, nitrate, and several metals, but was strongly inversely correlated with the relative abundance of *Gammaproteobacteria* (r^2^ = -0.76; Fig 3B and S5 Table). The relative abundance of *Gammaproteobacteria* varied significantly between dunes (p = 0.001). Dunes down wind of camel farms had a statistically higher mean percentage of *Gammaproteobacteria* than those that did not (p = 0.012; S5B Table). *Gammaproteobacteria* abundance, however, was not correlated with the moisture or organic matter content of the sands and was only weakly correlated with nitrate content and pH (S5 Table).

**Table 2:** Results of ANOVA and PERMANOVA analyses. P values less than 0.05 are bolded.

		Source	df	Sum of Squares	Mean Squares	F*	p-value
Alpha diversity	Factorial (between-dune) ANOVA	Dune	16	7.966	0.498	3.371	**0.001**
		Location	3	0.703	0.234	1.586	0.208
		Depth	3	0.196	0.065	0.441	0.725
		Residuals	40	5.91	0.148	-	-
	Nested (within-dune) ANOVA	Dune	16	7.966	0.498	7.745	**0.003**
		Location	25	4.591	0.184	2.856	0.063
		Depth	13	1.702	0.131	2.036	0.158
		Residuals	8	0.514	0.064	-	-
*Gammaproteobacteria* relative abundance	Factorial (between-dune) ANOVA	Dune	16	1.658	0.104	3.379	**0.001**
		Location	3	0.141	0.047	1.537	0.220
		Depth	3	0.006	0.002	0.064	0.979
		Residuals	40	1.227	0.031	-	-
	Nested (within-dune) ANOVA	Dune	16	1.658	0.104	5.799	**0.008**
		Location	25	1.113	0.045	2.491	0.091
		Depth	13	0.118	0.009	0.508	0.866
		Residuals	8	0.143	0.018	-	-
Microbial community composition	Between-dune PERMANOVA	Dune	13	10351	796.2	1.736	**0.045**
		Location	1	842.6	842.6	1.176	0.340
		Depth	0	-	-	-	-
		Residuals	37	19504	2594	-	-
	Within-dune PERMANOVA	Dune	16	19713	1232.1	2.722	**0.000**
		Location	25	14761	590.4	1.318	**0.046**
		Depth	20	9000.2	450.0	0.660	0.755
		Residuals	1	681.9	682.0	-	-

Similar to the trend observed in alpha diversity, total bacterial communities varied in composition between individual dunes regardless of sampling location on the dune (p = 0.045; Table 2) and dune size (p = 0.001; S5 Table). Unlike alpha diversity, bacterial community composition was found to vary significantly within an individual dune according to whether the community was sampled from the crest, middle, or base of the dune (p = 0.046), although communities from the crests of different dunes did not resemble one another, nor did those from the base or middle (p = 0.340). In addition, geographic distance between pairs of dunes did not correlate with the dissimilarity between their bacterial communities (p = 0.154).

**Fig 4:**
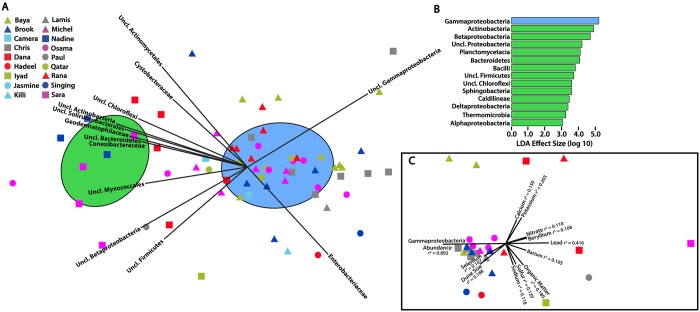
**A**. NMDS ordination of 16S rRNA gene-derived microbial community structure. Similarity profile analysis, an *a priori* statistical approach that uses permutation to identify groups of communities that are more dissimilar than expected by chance, identified two distinct clusters of communities. Ellipses represent the 95% confidence intervals around the centroid for each cluster (the spatial mean in NMDS space of the communities belonging to each cluster). Lines emanating from the centroids indicate to which cluster each community belongs. Bacterial families well-correlated with the ordination (r^2^ > 0.40) are displayed; vector length is proportional to the Pearson correlation coefficient for each family and vector direction corresponds to the direction of increasing abundance relative to the ordinated communities. Legend indicates the dune from which each ordinated community originated. Final 2-dimensional stress of the ordination is 0.12. **B**. Linear discriminant analysis (LDA) of bacterial classes indicates that the two clusters of microbial communities identified by similarity profile analysis are driven by the disparity between a high abundance of *Gammaproteobacteria* in one set of communities and more diverse population in the other set of communities. Only classes with effect size > 2.0 are displayed. **C**. NMDS ordination is based only on samples for which environmental parameters were measured. Parameters with r^2^ > 0.1 are displayed. Final 2-dimensional stress of the ordination is 0.07.

Hierarchical clustering revealed that the observed variance between bacterial communities was primarily due to differences in the abundances of *Gammaproteobacteria*, *Actinobacteria*, *Bacteroidetes*, *Chloroflexi*, and *Firmicutes* (Fig 2). This pattern was also identified by NMDS ordination (Fig 4). Two distinct clusters of bacterial communities were identified by similarity profile analysis, an *a priori* statistical approach that uses permutation to identify communities that are more dissimilar than expected by chance. Overlain in NMDS space, these two clusters appeared to be differentiated according to the abundance or rarity of Gammaproteobacteria and, correspondingly, the presence of a more or less diverse bacterial population (Fig 4A). Linear discriminant analysis (LDA) of the bacterial classes contributing to the observed clusters confirmed this result, as *Gammaproteobacteria* was the only class contributing significantly (defined here as having an LDA effect size greater than 2.0) to the larger cluster (which contained 54 out of 66 ordinated samples) (Fig 4B). Meanwhile, 13 classes originating from 6 phyla were significantly associated with communities present in the smaller cluster.

Modeling of bacterial community composition based on measured environmental parameters was performed using distance-based linear modeling. The best model included sodium, lead, potassium, and magnesium, explaining 38.1% of the community composition variance observed in the subset of samples for which environmental measurements were obtained. Pearson correlation to an NMDS ordination of these samples confirmed this result (Fig 4C). In addition, this analysis identified several other parameters, including organic matter and nitrate content that correlated weakly with the observed differences in bacterial community composition among samples.

### Shotgun Metagenomics Analysis

Analysis of two dune metagenomes (Nadine and Michel) revealed several notable differences in their phylogenetic and functional compositions. Michel had approximately 2.5 times more sequences annotated using the M5NR reference database than Nadine (see S6 Table for further details). Archaea were similarly abundant in both dunes (2.1–2.2%) and dominated by *Thaumarchaeota*, an archaeal group which can tolerate extreme environmental conditions. Representative members of this group have been shown to perform nitrification when ammonia concentrations in soils are low [54]. Bacteria dominated both metagenomes, but their relative abundance was lower in Michel (87.5%) than it was in Nadine (93.5%, S7 Table). In Nadine, the most abundant bacterial phyla were *Actinobacteria* (44.9%) and *Firmicutes* (24.8%), followed by *Proteobacteria* (13.0%) and *Bacteroidetes* (10.1%) (S7 Table). The relative abundances of these phyla were more evenly distributed in Michel (*Firmicutes* 26.7%, *Proteobacteria* 24.4.0%, *Bacteroidetes* 22.5%, and *Actinobacteria* 16.5%). Thus, the relative abundance of *Proteobacteria* was nearly twice as high in Michel as it was in Nadine. Also, the relative abundance of *Gammaproteobacteria* in Michel (11.8% of Bacteria) was more than three times that of Nadine (3.7%). The difference in relative abundance was even more dramatic for gut-associated enterics such as the genus *Escherichia* which was approximately 23 times more common in Michel (3.3%) than it was in Nadine (0.14%; S7 Table).

In Michel, located approximately 100 m downwind of a camel pen, the relative abundance of Eukaryotes was more than twice (9.5%) that found in Nadine (4.1%), with the latter being more than 1,000 m away and not immediately downwind of the pen (S1 Fig). More eukaryotic diversity was also detected in Michel, which was home to sequences from 233 families in 20 different phyla. Nadine on the other hand, harbored sequences from 97 families in 14 different phyla (S7 Table). The relative abundance of Fungi was similar: sequences annotated as *Ascomycota* were 7.9% and 6.6% of all Eukaryotic sequences, while *Basidiomycota* were 1.2% and 1.9% in Michel and Nadine respectively. The relative abundance of viruses, however, was 4 times greater in Michel (0.4%) than in Nadine (0.1%).

Overall, Nadine and Michel had similar functional abundances when compared using MG-RAST's Subsystems. The three most abundant Level 1 functions were carbohydrates (Nadine 12.8% and Michel 11.4%), protein metabolism (Nadine 11.1% and Michel 10.0%) and amino acids and derivatives (Nadine 9.6% and Michel 8.1%). Relative differences between the dunes were observed for potassium metabolism which was almost eight times more abundant in Michel (0.26%) than in Nadine (0.03%). The relative abundance of iron acquisition and metabolism was almost three times more in Michel (0.68%) than in Nadine (0.27%), and photosynthesis was almost two and a half times greater in Michel (0.12%) than in Nadine (0.05%; S4 Fig).

## Discussion

Very little is known about the microbiology of Barchan dunes [29], though stationary dunes [13, 23–28], and desert soils have been previously studied by others [12, 15–22, 25]. Our results provide the first metagenomic and deep 16S rRNA gene amplicon sequencing analysis of microbes from multiple mobile sand dunes in the same dune field and the first quantitative assessment of microbes in Qatari barchan dunes. We found that microbial community composition differed from dune to dune and from location to location in a given dune. Composition was influenced by size of the dune, but not by the depth from which the samples were taken. Less disturbed dunes had a greater relative abundance of *Firmicutes* and *Actinobacteria*, with a corresponding increase in genes encoding sporulation and dormancy. Dunes downwind of camel farms were dominated by *Proteobacteria*.

### Microbial enumeration

We found similar levels of bacteria on Qatari dune sands to those found on sands from Moroccan dunes [23] and on Middle Eastern desert sands [16] (S3 Table and Table 3). The number of CFUs from Qatari sands was also consistent with those of stationary Moroccan dunes [23], but more than those reported in Israeli dunes; [24]). In general we found fewer CFUs/g than have been reported in other desert soils [20, 55], perhaps due to desert soils being richer in organic matter than dune sands.

**Table 3:** Comparison of filter-based epifluorescent direct counts from Qatari dune (this work) with those of Middle Eastern and North African soils [16].

Topsoil Sample Location	Bacteria/ gram
Kuwait	1.0 x 10^5^
Morocco	5.7 x 10^4^
Qatar Dune*	5.3 x 10^4^
Saudi Arabia	4.4 x 10^4^
UAE	1.6 x 10^4^
Afghanistan	1.2 x 10^4^

### Shotgun Metagenomic and 16S rRNA Gene Amplicon Sequence Analyses

Our results from the analysis of 64 Qatari dune isolates were consistent with the 16S rRNA gene amplicons from other dunes [17, 22, 23] and a cold desert [55], specifically, aerobic bacteria from the phyla *Actinobacteria*, *Firmicutes*, and *Proteobacteria* were most frequently recovered [56]. Additionally, Gommeaux *et al*. [23] isolated several anaerobic *Bacteroidetes* species. Though the 16S rRNA gene sequences of both *Bacteroidetes* and *Chloroflexi* were amplified from sand DNA, we did not isolate any organisms from either of these two phyla. This was not surprising as many *Bacteroidetes* are fastidious [57] and *Chloroflexi* tend to be metabolic specialists (anaerobic phototrophs, halorespirers, thermophiles) [58], that would not have been isolated using the culturing conditions we employed.

Gommeaux *et al*. [23] also performed a direct analysis of 16S rRNA genes from genomic DNA extracted directly from sand, using a cloning and sequencing approach. While that work provided important insights into the bacterial diversity of their sand dune, their clone library analysis was limited (89 total), as it predated next generation sequencing efforts. More recently, others [13, 25, 26, 28] have performed deep 16S rRNA gene amplicon sequencing from dunes in the Gurbantunggut, Namib, Gobi and Taklamaken deserts, though they did not perform culture-based analysis on those sands. In addition to *Proteobacteria*, *Actinobacteria* and *Bacteroidetes*, Cyanobacteria were highly abundant in the Gurbantunggut sands due to the presence of BSCs [13, 26]. To our knowledge this work is the first to combine metagenomic and 16S rRNA gene amplicon analyses of dune microbial communities.

The Qatari dune bacterial communities characterized by 1) 16S rRNA gene amplicon analysis using MOTHUR, 2) shotgun metagenome analysis using phyloshop and 3) cultivation-based methods broadly agreed with each other (Table 4). These methods revealed that *Proteobacteria*, *Firmicutes*, and *Actinobacteria* were the most abundant phyla. However, MG-RAST analysis of the shotgun metagenomes suggested that *Bacteroidetes* were more than 10x more abundant than either the phyloshop or MOTHUR analyses.

**Table 4:** Comparison of the relative abundance of phyla between cultivation, deep 16S rRNA gene amplicon sequencing and metagenomics.

	Cultivation-based	16S rRNA Gene Amplicon Sequencing		Metagenomics (via Phyloshop)		Metagenomics (via MG-RAST)	
	Nadine	Michel	Nadine	Michel	Nadine	Michel	Nadine
*Actinobacteria*	58%	15.7%	36.4%	3.4%	12.6%	12.1%	36.7%
*Bacteroidetes*	N.D.	0.4%	3.4%	1.6%	2.4%	17.5%	6.0%
*Chloroflexi*	N.D.	0.3%	2.8%	0.3%	0.8%	1.4%	1.7%
*Firmicutes*	27%	2.0%	9.0%	10.7%	18.1%	22.1%	14.1%
*Proteobacteria*	15%	80.8%	47.9%	81.4%	59.1%	23.2%	16.0%

The high proportion of *Gammaproteobacteria* in the 16S rRNA gene deep-sequencing dataset (Fig 2) brings up the possibility of contamination during DNA extraction or sample preparation [59]. Although we cannot rule this out, the highly uneven distribution of *Gammaproteobacteria* across samples, and the strong correlation between *Gammaproteobacteria* percentage in dunes downwind of camel farms (P = 0.012), is not consistent with procedural contamination. Rather, it suggests that camel farms were a significant contributor to the percent of *Gammaproteobacteria* in dunes. The higher relative abundance of *Proteobacteria* in the 16S rRNA gene sequence dataset relative to the metagenomes may also be due in part to the higher 16S rRNA gene copy number in *Proteobacteria* relative to other detected phyla [60]. *Proteobacteria* were also dominant in the 16S rRNA gene amplicon sequences of other dune sands [26, 28]. Likewise, others have also observed that *Actinobacteria and Bacteroidetes* are abundant in desert soils [13, 15, 17, 25, 26, 28, 61]. In addition to these phyla, *Acidobacteria* were abundant in sand from the Tengger and Tataouine deserts [21, 22].

Many of the dunes we sampled had evidence of recreational human activities such as BBQ charcoal and food wrappers on their surfaces, as well as fecal matter that appeared to originate from resident dune organisms. Skinks (S*cincus mitranus*) were commonly observed on the avalanche face of Nadine, but not Michel. Beetles, which appeared to feed on fecal matter, were present on both Nadine and Michel. Michel had a much higher relative abundance of gut-associated enteric bacteria than Nadine in both the 16S rRNA gene amplicon-sequencing and the metagenomic datasets. The high relative abundance of enterics on Michel may have been the result of its proximity to a camel pen (approximately 100 m upwind of Michel; see S1 Fig). Other temporary camel pens were observed in the study area and their locations are indicated on S1 Fig. The observed dune-specific community patterns are likely impacted by the idiosyncratic nature of camel visitations to particular dunes and open access to any dune by the public, neither of which could be quantified or controlled. Indeed, the data in S5B Table demonstrate that there was a strong positive correlation between the proximity to camel pens and *Gamma*p*roteobacterial* relative abundance.

In addition to the often-dominating signature of *Proteobacteria*, each dune appeared to have a unique, though somewhat similar microbial community (Table 2). Despite measurement of numerous physical and chemical attributes, the only other factors playing a major role in community composition were dune size and the concentrations of iron and phosphorus. Dune size has been previously shown to correlate with the migration speeds of mobile barchan dunes [30, 62], which may impact both moisture content/distribution, and the aging of organic matter. Interestingly, MG-RAST analyses revealed that genes encoding iron acquisition were three times more abundant in Michel than in Nadine which also correlates with relative abundance of *Proteobacteria*, many of which are well-known for their redundant iron acquisition systems.

**Fig 5:**
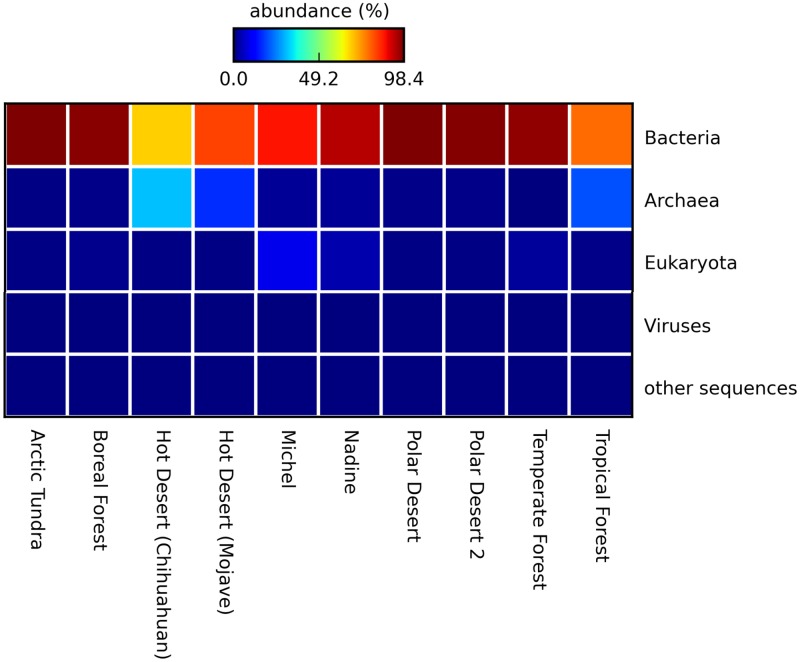
Relative abundance of domain-level sequences in the metagenomes of Nadine and Michel as compared with selected soil samples from Fierer *et al*., [15].

We compared our metagenomes with seven metagenomes described by Fierer *et al*. [15] that were available on MG-RAST (four desert and three forest soils, Fig 5) since no published sand metagenomes were available. In general, the relative abundance of Eukaryotes and Archea was higher in our samples, than it was in the soils studied by Fierer *et al*. [15]. The higher levels of Eukaryotes in our samples are consistent with anthropogenic impacts of camel farms. Pandit *et al*. [63] and Patel *et al*. [64] reported higher levels of Archaea in saline Indian desert soils. Archaea have previously been reported to be important components of saline soils [65], but were not as abundant in our dune sands despite the modestly alkaline pH and salinity.

While bacterial sequences dominated all of the metagenomes [15, 64], *Firmicutes* were more abundant in dunes we studied than in soils studied by Fierer *et al*. [15].

**Fig 6:**
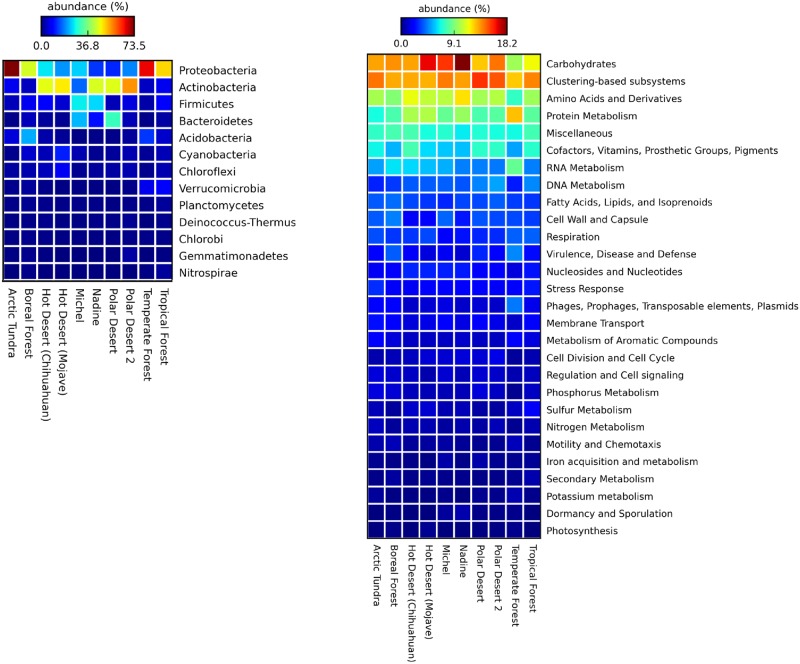
**A**. The relative abundance (based on Representative Hit Analysis in MG-RAST) of dominant bacterial phyla and **B**. MG-RAST Subsystems analysis (Level 1) of Nadine and Michel dunes, versus desert soils and non-desert soils studied by Fierer *et al*. [15]).

Further analysis of our metagenomes using MG-RAST's Subsystems revealed similar patterns for both dunes that were suggestive of microbial communities dominated by heterotrophic aerobic metabolism in a harsh environment (Fig 6B). Consistent with the increased relative abundance of *Firmicutes*, Dormancy and Sporulation sequences were more abundant in Qatari dunes than in the desert soils studied by Fierer *et al*. [15]. Members of this phylum are well known for forming desiccation-resistant endospores, which likely contributes to their ability to withstand the hot and dry climate of deserts and the shear forces experienced in mobile dunes [66]. Their greater abundance in Qatari dunes is not surprising given Qatar’s more extreme conditions where air temperatures regularly exceed 55°C, annual precipitation averages only 76.4 mm (1972–2014), and winds regularly exceed 5–30 km/h [67]. In contrast, precipitation is 3–4 times higher in the Mojave (330 mm) and Chihuahuan (235 mm) deserts that Fierer *et al*. [15] studied.

Also of note is the low relative abundance of phages and other mobile genetic elements in both our dunes and desert soils [15]. This may be due to the low biomass and spatial separation of bacteria (S2 Fig) which would reduce cell to cell contact opportunities and therefore the spread of such elements. Additionally, other factors such as limited nutrient availability and water holding capacity, which are known to affect horizontal gene transfer among microbes likely contributed. Others have also found relatively low abundance of virus-like particles in desert sands, though they found that many of the culturable bacteria harbored lysogens [17, 68, 69].

This work provides unique insights into the microbial communities of Qatari barchan dunes using both cultivation dependent and independent methods. We found that direct-on grain cell counts gave an average of 5.3 ± 0.4 x 10^5^ cells g^-1^ of sand. Cultured isolates belonged to the phyla *Actinobacteria* (58%), *Firmicutes* (27%) and *Proteobacteria* (15%). Analysis of 16S rRNA gene amplicons indicated that *Proteobacterial* genomic DNA dominated many of the dunes. It also revealed a dune-specific pattern of bacterial community composition that correlated with proximity to camel farms and dune size. The phylogenetic affiliation of sequences derived from the metagenome of the dune we studied most (Nadine) agreed closely at the taxonomic level of phylum with results obtained by culturing. These results differed somewhat from the results of the 16S rRNA gene amplicon sequencing, likely due to 16S rRNA gene operon copy number variation. Functional analysis using MG-RAST's Subsystems revealed similar patterns for both dunes that were suggestive of microbial communities dominated by heterotrophic aerobic spore-forming bacteria. Taken together, these results suggest that mobile barchan dunes in Qatar harbor unique microbial communities that are distinct from other soil environments, but that are readily impacted by anthropogenic activities.

## Supporting Information

S1 Fig Relative abundances of *Gammaproteobacteria* overlaid on a map of the sampled inland dunes. Crescents are scaled according to the horn-to-horn size of each dune as determined by measurement of images taken from Google Earth (11th February, 2015). Reported relative abundances represent the mean (from deep 16S rRNA gene amplicon analysis) for each portion of the dune face in the cases for which multiple samples were obtained. C = crest; M = middle; B = base; F = floor. Inset shows geographic location of mapped dunes within Qatar. Note that 7 sampled dunes from the eastern coast of Qatar are not represented in this map.
(TIFF)

S2 Fig A. Fluorescence micrograph of a sand grain treated with Syto9 fluorescent stain under 200X total magnification. Fluorescent dots were counted as single cells. B. Thirty-six grains incubated on 0.1X R2A solid medium.
(TIF)

S3 Fig Rarefaction curves for all samples included in the 16S rRNA gene amplicon deep sequencing analysis. All samples were rarefied to 15,710 sequences prior to analysis (vertical line). Legend indicates the dune of origin of each sample.
(TIFF)

S4 Fig MG-RAST metagenomic relative abundances of SEED Subsystems (Level 1) in Michel and Nadine. (TIF)

S1 Table Sample details and dune locations. Asterisks indicates samples removed from 16S rRNA gene amplicon sequencing analysis after rarefaction.
(DOCX)

S2 Table Measurements of abiotic environmental parameters. Moisture, organic matter, and total nitrogen are reported as %, all others as mg/kg sand. Sample key: dune name; location on dune face (C = crest, M = middle, B = base); sample number.
(DOCX)

S3 Table Number of colony forming units (± Standard Error) in a Qatari Barchan dune using different quantification methods. (DOCX)

S4 Table Identity and accession number of the closest BLAST match to the 16S rRNA gene sequences of isolates recovered from the single grain cultivations and serial dilutions. (DOCX)

S5 Table A. Results of multiple linear regression of significant environmental variables (p<0.05) to alpha diversity and *Gammaproteobacteria* relative abundance. B. Students Ttest demonstrated the mean *Gammaproteobacterial* abundance was greater for dunes located downwind of a camel farm (+), than for dunes that were not (-) (P = 0.012).
(DOCX)

S6 Table MG-RAST metagenomic sequencing details. (DOCX)

S7 Table MG-RAST metagenomic organism abundance analysis for Nadine and Michel dunes, based on Representative Hit Classification of the M5NR database. (DOCX)
