# Supplementary figures and images for: Microbial Characterization of Qatari Barchan Sand Dunes
Source: PLoS One. 2016 Sep 21;11(9):e0161836. doi: 10.1371/journal.pone.0161836 (PMC5031452; doi:10.1371/journal.pone.0161836)

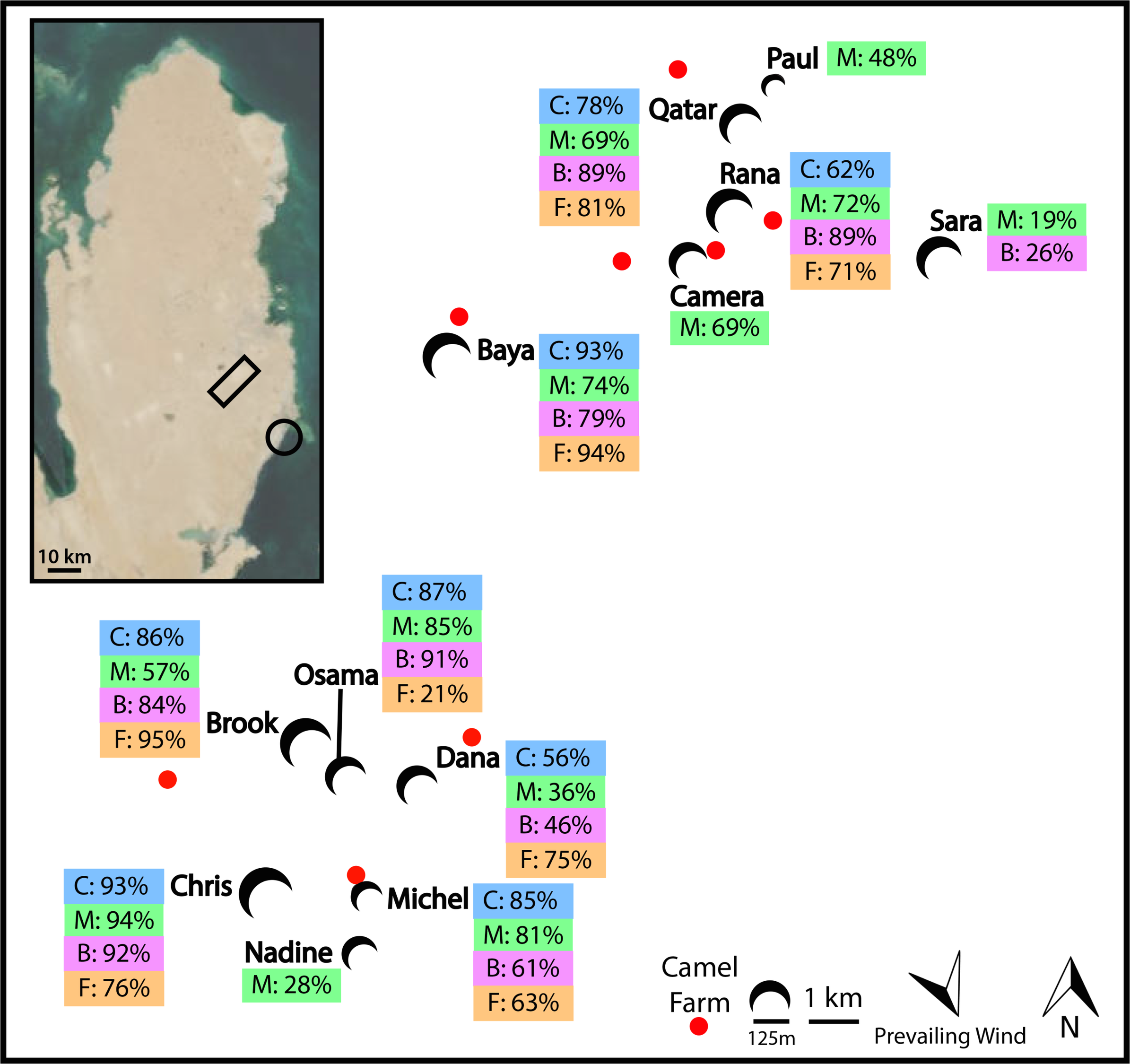

Supplement: S1 Fig — Crescents are scaled according to the horn-to-horn size of each dune as determined by measurement of images taken from Google Earth (11th February, 2015). Reported relative abundances represent the mean (from deep 16S rRNA gene amplicon analysis) for each portion of the dune face in the cases for which multiple samples were obtained. C = crest; M = middle; B = base; F = floor. Inset shows geographic location of mapped dunes within Qatar. Note that 7 sampled dunes from the eastern coast of Qatar are not represented in this map. (TIFF) [file pone.0161836.s001.tiff]

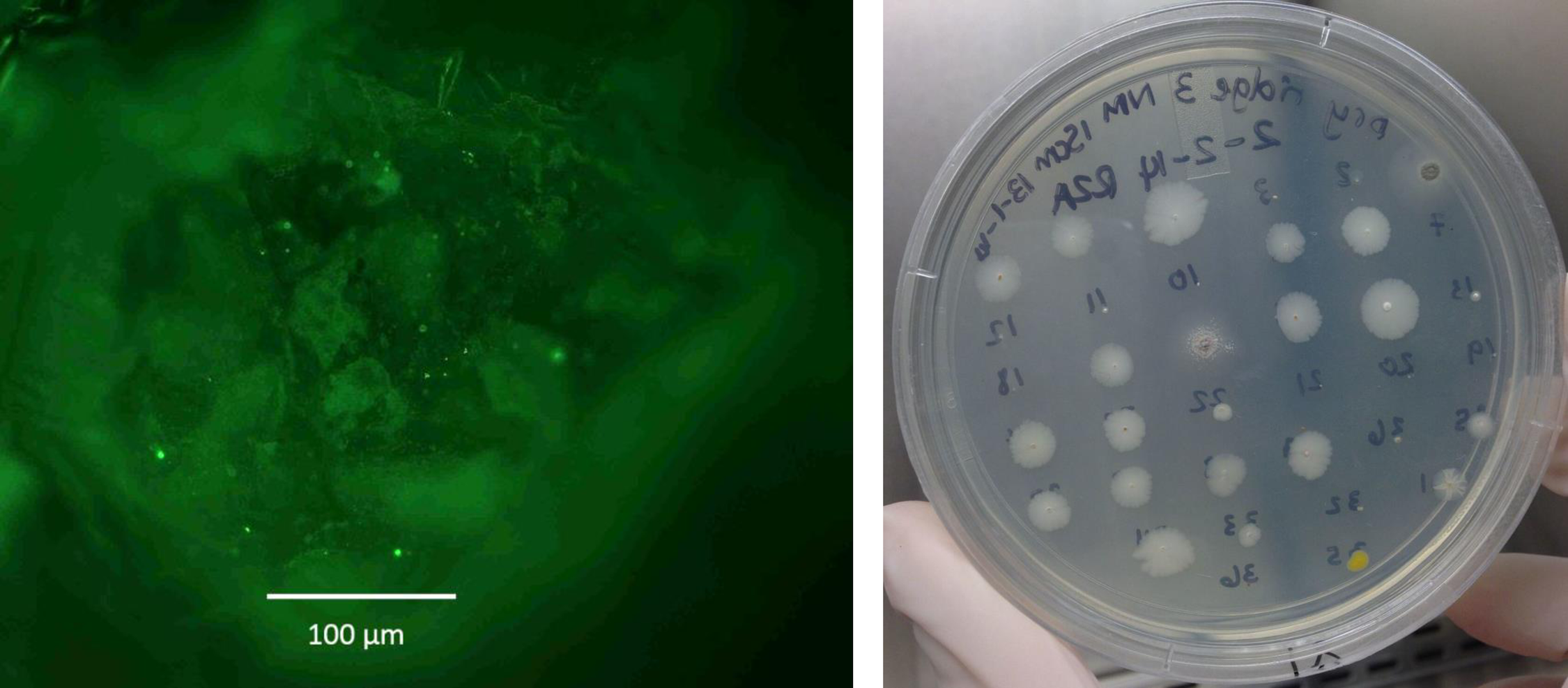

Supplement: S2 Fig — A. Fluorescence micrograph of a sand grain treated with Syto9 fluorescent stain under 200X total magnification. Fluorescent dots were counted as single cells. B. Thirty-six grains incubated on 0.1X R2A solid medium. (TIF) [file pone.0161836.s002.tif]

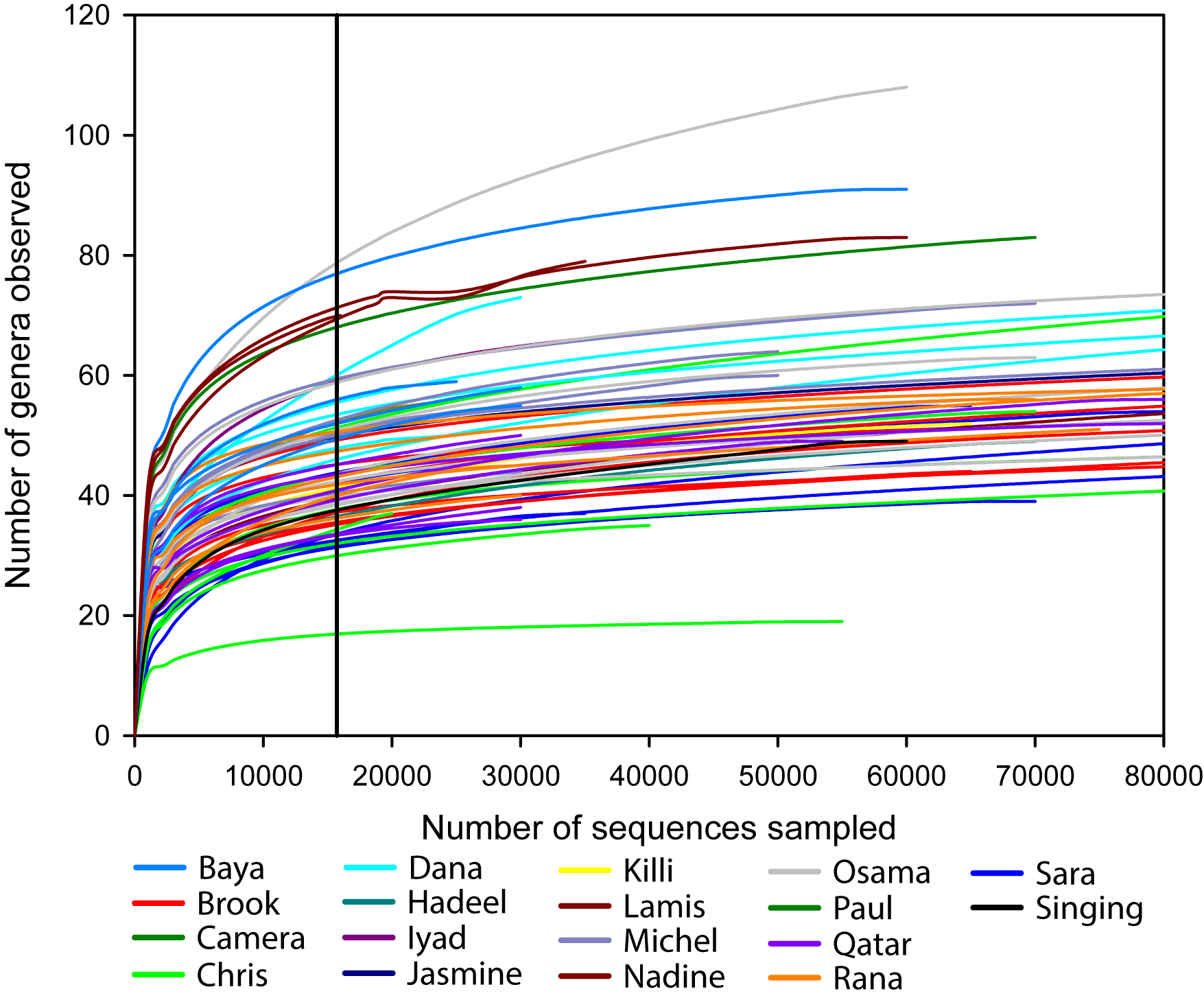

Supplement: S3 Fig — All samples were rarefied to 15,710 sequences prior to analysis (vertical line). Legend indicates the dune of origin of each sample. (TIFF) [file pone.0161836.s003.tiff]

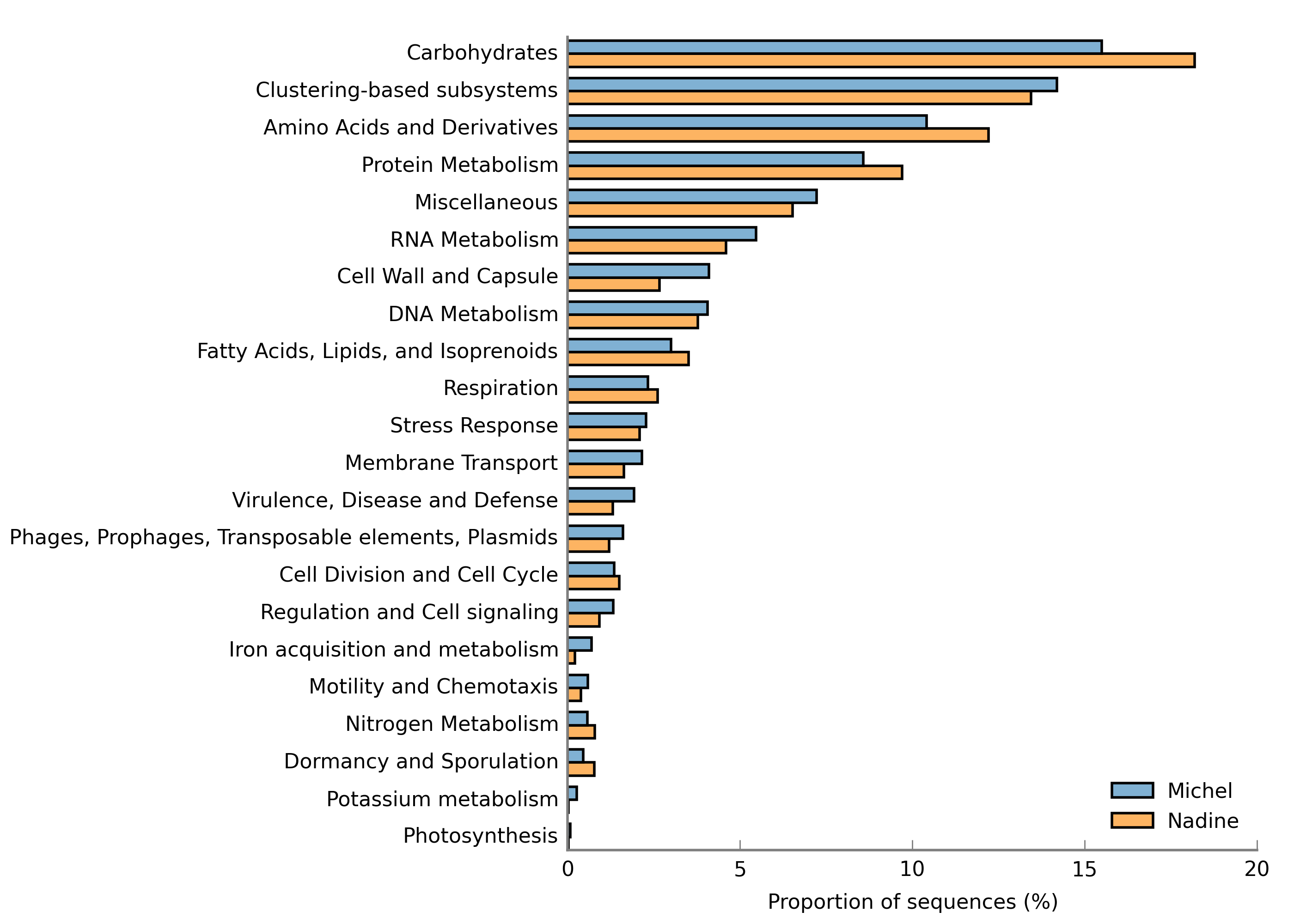

Supplement: S4 Fig — (TIF) [file pone.0161836.s004.tif]
