# Supplementary material for: Microbial Characterization of Qatari Barchan Sand Dunes
Source: PLoS One. 2016 Sep 21;11(9):e0161836. doi: 10.1371/journal.pone.0161836 (PMC5031452; doi:10.1371/journal.pone.0161836)
Supplement: S1 Table — Asterisks indicates samples removed from 16S rRNA gene amplicon sequencing analysis after rarefaction. (DOCX) [file pone.0161836.s005.docx]

**S1 Table.** Sample details and dune locations. Asterisks indicates samples removed from 16S deep-sequencing analysis after rarefaction.

| **16S**  **Code** | **Dune Name** | **Sample**  **Location** | **Depth** | **Collection Date** | **Coordinates Latitude** | **Coordinates Longitude** | **16S Fwd Barcode** | **16S Rev Barcode** | **No. Sequences** |
| --- | --- | --- | --- | --- | --- | --- | --- | --- | --- |
| **CC1** | Chris | Crest | 0-3 cm | 5/6/2011 | 25.01372 | 51.33351 | aggtg | tgaac | 51250 |
| **CC2** | Chris | Crest | 18-20 cm | 5/6/2011 | 25.01372 | 51.33351 | aggtg | atccg | 67413 |
| **CM1** | Chris | Middle | 0-5 cm | 5/6/2011 | 25.01372 | 51.33351 | aggtg | gaggt | 19684 |
| **CM2** | Chris | Middle | 13-15 cm | 5/6/2011 | 25.01372 | 51.33351 | aggtg | cctta | 38398 |
| **CB1** | Chris | Base | 0-5 cm | 5/6/2011 | 25.01372 | 51.33351 | aggtg | cccaa | 108821 |
| **CB2** | Chris | Base | 10-15 cm | 5/6/2011 | 25.01372 | 51.33351 | aggtg | gggtt | 110225 |
| **CF** | Chris | Floor | 0-1 cm | 5/6/2011 | 25.01372 | 51.33351 | aggtg | tttaa | 86733 |
|  |  |  |  |  |  |  |  |  |  |
| **OC1** | Osama | Crest | 0-3 cm | 5/6/2011 | 25.02156 | 51.33878 | ttggt | tgaac | 141650 |
| **OC2** | Osama | Crest | 12-15 cm | 5/6/2011 | 25.02156 | 51.33878 | ttggt | atccg | 137224 |
| **OM1** | Osama | Middle | 0-3 cm | 5/6/2011 | 25.02156 | 51.33878 | ttggt | gaggt | 61947 |
| **OM2** | Osama | Middle | 10-15 cm | 5/6/2011 | 25.02156 | 51.33878 | ttggt | cctta | 67253 |
| **OB1** | Osama | Base | 0-3 cm | 5/6/2011 | 25.02156 | 51.33878 | ttggt | cccaa | 170779 |
| **OB2** | Osama | Base | 10-15 cm | 5/6/2011 | 25.02156 | 51.33878 | ttggt | gggtt | 105717 |
| **OF** | Osama | Floor | 0-1 cm | 5/6/2011 | 25.02156 | 51.33878 | ttggt | tttaa | 129498 |
|  |  |  |  |  |  |  |  |  |  |
| **SC1** | Sara | Crest | 0-5 cm | 5/29/2011 | 25.06243 | 51.38939 | cttac | tgaac | 22* |
| **SC2** | Sara | Crest | 11-15 cm | 5/29/2011 | 25.06243 | 51.38939 | cttac | atccg | 16* |
| **SM1** | Sara | Middle | 0-5 cm | 5/29/2011 | 25.06243 | 51.38939 | cttac | gaggt | 55865 |
| **SM2** | Sara | Middle | 11-15 cm | 5/29/2011 | 25.06243 | 51.38939 | cttac | cctta | 22879 |
| **SB1** | Sara | Base | 0-5 cm | 5/29/2011 | 25.06243 | 51.38939 | cttac | cccaa | 29759 |
| **SB2** | Sara | Base | 16-20 cm | 5/29/2011 | 25.06243 | 51.38939 | cttac | gggtt | 28034 |
|  |  |  |  |  |  |  |  |  |  |
| **MC1** | Michel | Crest | 0-3 cm | 6/22/2011 | 25.01361 | 51.34109 | tccga | tgaac | 48608 |
| **MC2** | Michel | Crest | 22-24 cm | 6/22/2011 | 25.01361 | 51.34109 | tccga | atccg | 24396 |
| **MM1** | Michel | Middle | 0-2 cm | 6/22/2011 | 25.01361 | 51.34109 | tccga | gaggt | 68193 |
| **MM2** | Michel | Middle | 9-12 cm | 6/22/2011 | 25.01361 | 51.34109 | tccga | cctta | 29581 |
| **MB1** | Michel | Base | 0-5 cm | 6/22/2011 | 25.01361 | 51.34109 | tccga | cccaa | 339229 |
| **MB2** | Michel | Base | 12-15 cm | 6/22/2011 | 25.01361 | 51.34109 | tccga | gggtt | 3323* |
| **MF** | Michel | Floor | 0-1 cm | 6/22/2011 | 25.01361 | 51.34109 | tccga | tttaa | 47018 |
|  |  |  |  |  |  |  |  |  |  |
| **NM1** | Nadine | Middle |  |  | 25.008878 | 51.340576 | ggttg | gaggt | 70165 |
| **NM2** | Nadine | Middle |  |  | 25.008878 | 51.340576 | ggttg | tgaac | 27746 |
| **NM3** | Nadine | Middle |  |  | 25.008878 | 51.340576 | ggttg | aacca | 144379 |
|  |  |  |  |  |  |  |  |  |  |
| **BrC1** | Brook | Crest | 0-3 cm | 6/27/2011 | 25.02463 | 51.33647 | gaact | tgaac | 63742 |
| **BrC2** | Brook | Crest | 10-15 cm | 6/27/2011 | 25.02463 | 51.33647 | gaact | atccg | 95350 |
| **BrM1** | Brook | Middle | 0-4 cm | 6/27/2011 | 25.02463 | 51.33647 | gaact | gaggt | 84972 |
| **BrM2** | Brook | Middle | 11-15 cm | 6/27/2011 | 25.02463 | 51.33647 | gaact | cctta | 170554 |
| **BrB1** | Brook | Base | 0-3 cm | 6/27/2011 | 25.02463 | 51.33647 | gaact | cccaa | 145240 |
| **BrB2** | Brook | Base | 10-13 cm | 6/27/2011 | 25.02463 | 51.33647 | gaact | gggtt | 132046 |
| **BrF** | Brook | Floor | 0-1 cm | 6/27/2011 | 25.02463 | 51.33647 | gaact | tttaa | 96720 |
|  |  |  |  |  |  |  |  |  |  |
| **A** | Anthony | Middle | 10-15cm | 9/23/2011 | 25.01822 | 51.27913 | ccaac | tttaa | 9403* |
|  |  |  |  |  |  |  |  |  |  |
| **BaC1** | Baya | Crest | 0-5 cm | 9/23/2011 | 25.05379 | 51.34764 | acagt | tgaac | 65480 |
| **BaC2** | Baya | Crest | 15-20 cm | 9/23/2011 | 25.05379 | 51.34764 | acagt | atccg | 105896 |
| **BaM1** | Baya | Middle | 0-5 cm | 9/23/2011 | 25.05379 | 51.34764 | acagt | gaggt | 77063 |
| **BaM2** | Baya | Middle | 20-25 cm | 9/23/2011 | 25.05379 | 51.34764 | acagt | cctta | 2362* |
| **BaB1** | Baya | Base | 0-5 cm | 9/23/2011 | 25.05379 | 51.34764 | acagt | cccaa | 61831 |
| **BaB2** | Baya | Base | 20-25 cm | 9/23/2011 | 25.05379 | 51.34764 | acagt | gggtt | 99338 |
| **BaF** | Baya | Floor | 0-1 cm | 9/23/2011 | 25.05379 | 51.34764 | acagt | tttaa | 31682 |
|  |  |  |  |  |  |  |  |  |  |
| **C** | Camera | Middle | 10-15cm | 9/23/2011 | 25.06165 | 51.36832 | ccaac | cccaa | 23310 |
| **F** | Feras | Middle | 10-15cm | 10/19/2011 | 25.04109 | 51.35528 | ccaac | gggtt | 8020* |
| **DC1** | Dana | Crest | 0-5 cm | 10/19/2011 | 25.02126 | 51.34473 | cactg | tgaac | 81564 |
| **DC2** | Dana | Crest | 15-20 cm | 10/19/2011 | 25.02126 | 51.34473 | cactg | atccg | 100903 |
| **DM1** | Dana | Middle | 0-5 cm | 10/19/2011 | 25.02126 | 51.34473 | cactg | gaggt | 6952* |
| **DM2** | Dana | Middle | 10-15 cm | 10/19/2011 | 25.02126 | 51.34473 | cactg | cctta | 159375 |
| **DB1** | Dana | Base | 0-5 cm | 10/19/2011 | 25.02126 | 51.34473 | cactg | cccaa | 5967* |
| **DB2** | Dana | Base | 10-15 cm | 10/19/2011 | 25.02126 | 51.34473 | cactg | gggtt | 22693 |
| **DF** | Dana | Floor | 0-1 cm | 10/19/2011 | 25.02126 | 51.34473 | cactg | tttaa | 27924 |
|  |  |  |  |  |  |  |  |  |  |
| **QC 1** | Qatar | Crest | 0-5 cm | 11/4/2011 | 25.07383 | 51.37327 | gtgac | tgaac | 29126 |
| **QC 2** | Qatar | Crest | 10-15 cm | 11/4/2011 | 25.07383 | 51.37327 | gtgac | atccg | 29811 |
| **QM 1** | Qatar | Middle | 0-5 cm | 11/4/2011 | 25.07383 | 51.37327 | gtgac | gaggt | 7867* |
| **QM 2** | Qatar | Middle | 10-15 cm | 11/4/2011 | 25.07383 | 51.37327 | gtgac | cctta | 50779 |
| **QB 1** | Qatar | Base | 0-5 cm | 11/4/2011 | 25.07383 | 51.37327 | gtgac | cccaa | 29300 |
| **QB 2** | Qatar | Base | 10-15 cm | 11/4/2011 | 25.07383 | 51.37327 | gtgac | gggtt | 75948 |
| **QF** | Qatar | Floor | 0-1 cm | 11/4/2011 | 25.07383 | 51.37327 | gtgac | tttaa | 77711 |
|  |  |  |  |  |  |  |  |  |  |
| **RC1** | Rana | Crest | 0-5 cm | 11/4/2011 | 25.06641 | 51.37168 | tgtca | tgaac | 65378 |
| **RC2** | Rana | Crest | 10-15 cm | 11/4/2011 | 25.06641 | 51.37168 | tgtca | atccg | 74644 |
| **RM1** | Rana | Middle | 0-5 cm | 11/4/2011 | 25.06641 | 51.37168 | tgtca | gaggt | 85735 |
| **RM2** | Rana | Middle | 10-15 cm | 11/4/2011 | 25.06641 | 51.37168 | tgtca | cctta | 80012 |
| **RB1** | Rana | Base | 0-5 cm | 11/4/2011 | 25.06641 | 51.37168 | tgtca | cccaa | 29015 |
| **RB2** | Rana | Base | 10-15 cm | 11/4/2011 | 25.06641 | 51.37168 | tgtca | gggtt | 7070* |
| **RF** | Rana | Floor | 0-1 cm | 11/4/2011 | 25.06641 | 51.37168 | tgtca | tttaa | 27091 |
|  |  |  |  |  |  |  |  |  |  |
| **G** | Girlie | Middle | 10-15 cm | 11/4/2011 | 24.77939 | 51.45626 | ccaac | gaggt | 14174* |
|  |  |  |  |  |  |  |  |  |  |
| **H** | Hadeel | Middle | 10-15 cm | 11/4/2011 | 24.76487 | 51.44834 | ccaac | tgaac | 9240* |
| **Hplant** | Hadeel | Middle under grass | 0-3 cm | 11/4/2011 | 24.76487 | 51.44834 | acagt | aacca | 68528 |
|  |  |  |  |  |  |  |  |  |  |
| **I** | Iyad | Middle | 10-15 cm | 11/4/2011 | ? | ? | ccaac | cctta | 54918 |
|  |  |  |  |  |  |  |  |  |  |
| **J** | Jasmine | Middle | 10-15 cm | 11/4/2011 | 24.76620 | 51.44463 | ccaac | atccg | 123240 |
|  |  |  |  |  |  |  |  |  |  |
| **K** | Killi | Middle | 10-15 cm | 11/4/2011 | 24.77639 | 51.44905 | ccaac | aacca | 63622 |
|  |  |  |  |  |  |  |  |  |  |
| **L** | Lamis | Middle | 10-15 cm | 11/4/2011 | 24.85263 | 51.48278 | ttggt | aacca | 98894 |
|  |  |  |  |  |  |  |  |  |  |
| **N** | Natalie | Middle | 10-15 cm | 11/4/2011 | 25.08557 | 51.38418 | aggtg | aacca | 53606 |
|  |  |  |  |  |  |  |  |  |  |
| **P** | Perez | Middle | 10-15 cm | 11/4/2011 | 24.83340 | 51.48883 | cttac | aacca | 1000* |
|  |  |  |  |  |  |  |  |  |  |
| **Pa** | Paul | Middle | 10-15 cm | 11/4/2011 | 25.07690 | 51.37697 | gaact | aacca | 69446 |
|  |  |  |  |  |  |  |  |  |  |
| **S** | Singing | Middle | 10-15 cm | 11/4/2011 | 24.77265 | 51.45235 | tccga | aacca | 55831 |
