# Supplementary material for: Microbial Characterization of Qatari Barchan Sand Dunes
Source: PLoS One. 2016 Sep 21;11(9):e0161836. doi: 10.1371/journal.pone.0161836 (PMC5031452; doi:10.1371/journal.pone.0161836)
Supplement: S2 Table — Moisture, organic matter, and total nitrogen are reported as %, all others as mg/kg sand. Sample key: dune name; location on dune face (C = crest, M = middle, B = base); sample number. (DOCX) [file pone.0161836.s006.docx]

**S2 Table.** Measurements of abiotic environmental parameters. Moisture, organic matter, and total nitrogen are reported as %, all others as mg/kg sand. Sample key: dune name; location on dune face (C=crest, M=middle, B=base); sample number.

|  | **Dune Size (m)** |  | **Moist (wt %)** | **pH** | **Organic Matter (wt %)** | **Total Nitrogen (wt %)** | **Ammonia (mg/kg)** | **Nitrate (mg/kg)** | **Aluminum (mg/kg)** | **Arsenic (mg/kg)** | **Boron (mg/kg)** | **Barium (mg/kg)** | **Beryllium (mg/kg)** | **Calcium (mg/kg)** | **Cadmium (mg/kg)** | **Cobalt (mg/kg)** | **Chromium (mg/kg)** | **Copper (mg/kg)** | **Iron (mg/kg)** | **Potassium (mg/kg)** | **Lithium (mg/kg)** | **Magnesium (mg/kg)** | **Manganese (mg/kg)** | **Molybdenum (mg/kg)** | **Sodium (mg/kg)** | **Nickel (mg/kg)** | **Phosphorous (mg/kg)** | **Lead (mg/kg)** | **Sulfur (mg/kg)** | **Selenium (mg/kg)** | **Titanium (mg/kg)** | **Vanadium (mg/kg)** | **Zinc (mg/kg)** |
| --- | --- | --- | --- | --- | --- | --- | --- | --- | --- | --- | --- | --- | --- | --- | --- | --- | --- | --- | --- | --- | --- | --- | --- | --- | --- | --- | --- | --- | --- | --- | --- | --- | --- |
|  |  |  |  |  |  |  |  |  |  |  |  |  |  |  |  |  |  |  |  |  |  |  |  |  |  |  |  |  |  |  |  |  |  |
| **Baya B2** | 2.55E+02 | | 1.20E-01 | 9.28E+00 | 0.00E+00 | 4.40E-01 | 0.00E+00 | 4.40E-01 | 1.72E+03 | 7.10E-01 | 1.36E+01 | 1.72E+01 | 0.00E+00 | 1.75E+05 | 0.00E+00 | 1.31E+00 | 6.75E+00 | 4.45E+00 | 1.70E+03 | 4.23E+02 | 3.82E+00 | 6.13E+03 | 5.22E+01 | 2.00E-01 | 4.31E+02 | 6.88E+00 | 2.05E+02 | 1.33E+00 | 4.68E+02 | 1.33E+00 | 3.11E+01 | 4.32E+00 | 3.04E+00 |
| **Baya C1** | 2.55E+02 | | 1.20E-01 | 9.48E+00 | 4.00E-02 | 1.02E+00 | 0.00E+00 | 1.02E+00 | 2.34E+03 | 1.11E+00 | 1.22E+01 | 1.87E+01 | 0.00E+00 | 2.09E+05 | 0.00E+00 | 1.78E+00 | 1.09E+01 | 4.24E+00 | 2.53E+03 | 4.64E+02 | 4.59E+00 | 7.01E+03 | 7.44E+01 | 3.50E-01 | 5.01E+02 | 8.87E+00 | 2.25E+02 | 1.29E+00 | 5.51E+02 | 1.28E+00 | 5.49E+01 | 6.03E+00 | 3.40E+00 |
| **Baya C2** | 2.55E+02 | | 1.30E-01 | 9.26E+00 | 7.00E-02 | 7.80E-01 | 0.00E+00 | 7.80E-01 | 2.29E+03 | 1.57E+00 | 1.29E+01 | 1.97E+01 | 0.00E+00 | 2.19E+05 | 0.00E+00 | 1.79E+00 | 9.31E+00 | 6.48E+00 | 2.40E+03 | 4.97E+02 | 4.81E+00 | 7.34E+03 | 7.12E+01 | 4.10E-01 | 5.18E+02 | 9.01E+00 | 2.27E+02 | 1.50E+00 | 5.72E+02 | 1.05E+00 | 4.85E+01 | 6.03E+00 | 3.95E+00 |
| **Baya M2** | 2.55E+02 | | 1.30E-01 | 9.36E+00 | 1.20E-01 | 1.28E+00 | 0.00E+00 | 1.28E+00 | 2.89E+03 | 1.54E+00 | 1.29E+01 | 2.34E+01 | 1.00E-02 | 2.39E+05 | 0.00E+00 | 2.16E+00 | 1.52E+01 | 5.72E+00 | 3.19E+03 | 5.25E+02 | 5.59E+00 | 8.37E+03 | 9.04E+01 | 5.30E-01 | 5.82E+02 | 1.10E+01 | 2.76E+02 | 2.04E+00 | 6.30E+02 | 1.12E+00 | 7.02E+01 | 7.28E+00 | 5.15E+00 |
| **Brook B2** | 3.45E+02 | | 6.00E-02 | 9.53E+00 | 0.00E+00 | 1.30E-01 | 0.00E+00 | 1.30E-01 | 1.27E+03 | 0.00E+00 | 1.13E+01 | 1.35E+01 | 0.00E+00 | 1.33E+05 | 0.00E+00 | 1.05E+00 | 4.93E+00 | 5.96E+00 | 1.20E+03 | 3.50E+02 | 2.87E+00 | 5.10E+03 | 3.50E+01 | 2.30E-01 | 3.20E+02 | 5.34E+00 | 1.61E+02 | 1.40E+00 | 3.88E+02 | 9.80E-01 | 2.10E+01 | 3.01E+00 | 3.71E+00 |
| **Brook C1** | 3.45E+02 | | 1.00E-01 | 9.46E+00 | 9.00E-02 | 9.40E-01 | 0.00E+00 | 9.40E-01 | 2.42E+03 | 6.60E-01 | 1.21E+01 | 1.99E+01 | 0.00E+00 | 1.74E+05 | 0.00E+00 | 1.90E+00 | 9.90E+00 | 5.81E+00 | 2.77E+03 | 4.60E+02 | 4.65E+00 | 7.12E+03 | 7.48E+01 | 3.90E-01 | 4.50E+02 | 9.47E+00 | 2.09E+02 | 1.71E+00 | 4.68E+02 | 1.16E+00 | 6.32E+01 | 6.23E+00 | 5.28E+00 |
| **Brook C2** | 3.45E+02 | | 8.00E-02 | 9.42E+00 | 4.00E-02 | 1.03E+00 | 0.00E+00 | 1.03E+00 | 2.44E+03 | 6.70E-01 | 1.04E+01 | 1.98E+01 | 0.00E+00 | 1.93E+05 | 0.00E+00 | 1.85E+00 | 9.18E+00 | 6.41E+00 | 2.68E+03 | 4.80E+02 | 4.76E+00 | 7.06E+03 | 7.08E+01 | 4.70E-01 | 5.07E+02 | 9.48E+00 | 1.97E+02 | 2.07E+00 | 5.19E+02 | 1.03E+00 | 4.36E+01 | 4.95E+00 | 4.56E+00 |
| **Brook M2** | 3.45E+02 | | 1.20E-01 | 9.50E+00 | 0.00E+00 | 5.00E-01 | 0.00E+00 | 5.00E-01 | 2.76E+03 | 1.30E-01 | 1.10E+01 | 2.36E+01 | 0.00E+00 | 1.76E+05 | 0.00E+00 | 1.99E+00 | 1.04E+01 | 5.88E+00 | 2.86E+03 | 5.20E+02 | 5.31E+00 | 7.60E+03 | 8.15E+01 | 2.70E-01 | 4.89E+02 | 1.07E+01 | 2.07E+02 | 1.62E+00 | 4.85E+02 | 1.19E+00 | 6.42E+01 | 5.77E+00 | 5.83E+00 |
| **Chris B2** | 4.27E+02 | | 1.70E-01 | 9.69E+00 | 0.00E+00 | 3.80E-01 | 6.00E-02 | 3.20E-01 | 2.09E+03 | 4.90E-01 | 1.07E+01 | 2.02E+01 | 0.00E+00 | 1.69E+05 | 0.00E+00 | 1.61E+00 | 7.39E+00 | 4.83E+00 | 2.41E+03 | 4.61E+02 | 4.32E+00 | 6.84E+03 | 6.21E+01 | 3.30E-01 | 4.74E+02 | 8.59E+00 | 1.98E+02 | 1.69E+00 | 5.21E+02 | 1.22E+00 | 3.65E+01 | 4.52E+00 | 3.80E+00 |
| **Chris C1** | 4.27E+02 | | 1.40E-01 | 9.43E+00 | 6.00E-02 | 1.10E+00 | 2.50E-01 | 8.50E-01 | 2.70E+03 | 3.00E-01 | 9.24E+00 | 2.00E+01 | 0.00E+00 | 2.07E+05 | 0.00E+00 | 1.95E+00 | 1.17E+01 | 5.63E+00 | 2.82E+03 | 4.86E+02 | 5.18E+00 | 7.63E+03 | 8.21E+01 | 3.80E-01 | 5.26E+02 | 1.00E+01 | 2.02E+02 | 1.89E+00 | 5.28E+02 | 1.13E+00 | 4.72E+01 | 5.02E+00 | 4.00E+00 |
| **Chris C2** | 4.27E+02 | | 1.40E-01 | 9.66E+00 | 2.00E-02 | 1.21E+00 | 5.30E-01 | 6.80E-01 | 2.09E+03 | 0.00E+00 | 8.44E+00 | 1.99E+01 | 0.00E+00 | 1.36E+05 | 0.00E+00 | 1.55E+00 | 7.07E+00 | 6.11E+00 | 2.18E+03 | 4.33E+02 | 4.03E+00 | 6.05E+03 | 5.65E+01 | 2.50E-01 | 3.63E+02 | 8.22E+00 | 1.77E+02 | 1.78E+00 | 3.75E+02 | 1.03E+00 | 3.65E+01 | 4.12E+00 | 4.61E+00 |
| **Chris M2** | 4.27E+02 | | 1.40E-01 | 9.53E+00 | 0.00E+00 | 5.30E-01 | 1.20E-01 | 4.10E-01 | 1.87E+03 | 1.10E-01 | 8.96E+00 | 1.85E+01 | 0.00E+00 | 1.66E+05 | 0.00E+00 | 1.55E+00 | 6.68E+00 | 5.22E+00 | 2.21E+03 | 4.41E+02 | 4.14E+00 | 6.75E+03 | 5.39E+01 | 4.40E-01 | 4.50E+02 | 8.22E+00 | 1.83E+02 | 1.43E+00 | 4.59E+02 | 1.20E+00 | 2.84E+01 | 3.91E+00 | 4.59E+00 |
| **Dana C1** | 1.28E+02 | | 1.20E-01 | 9.29E+00 | 2.00E-02 | 2.97E+00 | 2.30E-01 | 2.74E+00 | 2.39E+03 | 3.80E-01 | 7.88E+00 | 2.07E+01 | 0.00E+00 | 2.20E+05 | 0.00E+00 | 2.02E+00 | 1.44E+01 | 5.62E+00 | 2.90E+03 | 4.94E+02 | 4.82E+00 | 8.56E+03 | 7.96E+01 | 5.70E-01 | 5.63E+02 | 1.02E+01 | 1.97E+02 | 2.08E+00 | 6.13E+02 | 1.22E+00 | 4.46E+01 | 4.73E+00 | 3.82E+00 |
| **Dana C2** | 1.28E+02 | | 8.00E-02 | 9.21E+00 | 1.00E-02 | 2.30E+00 | 7.30E-01 | 1.57E+00 | 1.64E+03 | 3.10E-01 | 1.02E+01 | 1.46E+01 | 0.00E+00 | 2.00E+05 | 0.00E+00 | 1.44E+00 | 6.65E+00 | 4.27E+00 | 1.77E+03 | 5.70E+02 | 3.72E+00 | 6.89E+03 | 5.00E+01 | 3.60E-01 | 4.81E+02 | 7.14E+00 | 2.14E+02 | 1.53E+00 | 5.62E+02 | 9.90E-01 | 2.56E+01 | 3.97E+00 | 2.17E+00 |
| **Feras M** | 2.85E+02 | | 8.00E-02 | 9.48E+00 | 4.00E-02 | 1.15E+00 | 0.00E+00 | 1.15E+00 | 3.54E+03 | 3.40E-01 | 9.97E+00 | 2.58E+01 | 2.00E-02 | 2.63E+05 | 0.00E+00 | 2.48E+00 | 1.15E+01 | 6.23E+00 | 3.61E+03 | 5.65E+02 | 6.40E+00 | 9.12E+03 | 1.08E+02 | 3.80E-01 | 6.15E+02 | 1.28E+01 | 2.37E+02 | 2.01E+00 | 6.15E+02 | 1.12E+00 | 6.69E+01 | 6.53E+00 | 5.68E+00 |
| **Hadeel M** | 2.48E+02 | | 9.00E-02 | 8.83E+00 | 6.00E-02 | 5.70E-01 | 0.00E+00 | 5.70E-01 | 1.82E+03 | 3.10E-01 | 1.10E+01 | 1.75E+01 | 0.00E+00 | 7.94E+04 | 0.00E+00 | 1.03E+00 | 5.52E+00 | 3.87E+00 | 1.70E+03 | 3.95E+02 | 3.42E+00 | 5.57E+03 | 4.72E+01 | 1.90E-01 | 5.49E+02 | 6.28E+00 | 1.36E+02 | 8.70E-01 | 5.79E+02 | 1.20E+00 | 5.08E+01 | 4.12E+00 | 2.53E+00 |
| **Iyad M** | Not measured | | 2.60E-01 | 8.86E+00 | 1.70E-01 | 6.90E-01 | 0.00E+00 | 6.90E-01 | 2.33E+03 | 5.90E-01 | 1.06E+01 | 2.10E+01 | 0.00E+00 | 1.22E+05 | 0.00E+00 | 1.54E+00 | 6.09E+00 | 5.05E+00 | 2.29E+03 | 4.84E+02 | 5.22E+00 | 6.84E+03 | 6.20E+01 | 2.20E-01 | 9.29E+02 | 8.85E+00 | 1.48E+02 | 1.24E+00 | 1.90E+03 | 1.16E+00 | 5.44E+01 | 4.67E+00 | 3.83E+00 |
| **Lamis M** | 2.98E+02 | | 9.00E-02 | 8.56E+00 | 1.20E-01 | 1.42E+00 | 1.50E-01 | 1.27E+00 | 3.41E+03 | 2.40E-01 | 1.07E+01 | 2.67E+01 | 1.00E-02 | 1.28E+05 | 0.00E+00 | 2.42E+00 | 3.69E+01 | 5.35E+00 | 3.66E+03 | 5.48E+02 | 6.36E+00 | 9.54E+03 | 1.27E+02 | 3.10E-01 | 7.86E+02 | 1.30E+01 | 1.69E+02 | 1.86E+00 | 4.46E+02 | 1.14E+00 | 1.34E+02 | 7.45E+00 | 6.84E+00 |
| **Michel B2** | 5.08E+01 | | 1.00E-01 | 9.25E+00 | 6.00E-02 | 2.25E+00 | 3.00E-02 | 2.22E+00 | 1.84E+03 | 1.48E+00 | 8.64E+00 | 1.68E+01 | 0.00E+00 | 2.05E+05 | 0.00E+00 | 1.61E+00 | 6.64E+00 | 4.31E+00 | 2.25E+03 | 4.33E+02 | 4.21E+00 | 7.49E+03 | 5.94E+01 | 4.10E-01 | 5.75E+02 | 8.01E+00 | 2.21E+02 | 2.04E+00 | 5.61E+02 | 1.27E+00 | 2.70E+01 | 3.85E+00 | 2.18E+00 |
| **Michel C2** | 5.08E+01 | | 1.00E-01 | 9.43E+00 | 5.00E-02 | 1.78E+00 | 0.00E+00 | 1.78E+00 | 1.97E+03 | 1.20E+00 | 9.99E+00 | 1.74E+01 | 0.00E+00 | 2.30E+05 | 0.00E+00 | 1.81E+00 | 9.29E+00 | 6.69E+00 | 3.12E+03 | 4.37E+02 | 4.36E+00 | 8.07E+03 | 6.96E+01 | 8.00E-01 | 6.44E+02 | 9.27E+00 | 2.54E+02 | 2.27E+00 | 8.25E+02 | 1.26E+00 | 3.49E+01 | 4.68E+00 | 5.62E+00 |
| **Michel M2** | 5.08E+01 | | 1.20E-01 | 9.45E+00 | 2.00E-02 | 2.49E+00 | 0.00E+00 | 2.49E+00 | 2.07E+03 | 1.18E+00 | 7.54E+00 | 1.83E+01 | 0.00E+00 | 2.27E+05 | 0.00E+00 | 1.81E+00 | 6.49E+00 | 4.92E+00 | 2.67E+03 | 4.44E+02 | 4.52E+00 | 7.96E+03 | 6.91E+01 | 7.00E-01 | 6.11E+02 | 9.00E+00 | 2.07E+02 | 2.06E+00 | 6.98E+02 | 1.51E+00 | 3.48E+01 | 4.19E+00 | 4.17E+00 |
| **Nadine B1** | 9.25E+01 | | 1.20E-01 | 9.33E+00 | 5.00E-02 | 1.39E+00 | 0.00E+00 | 1.39E+00 | 3.33E+03 | 0.00E+00 | 7.50E+00 | 2.40E+01 | 2.00E-02 | 1.69E+05 | 0.00E+00 | 2.26E+00 | 8.55E+00 | 5.57E+00 | 3.33E+03 | 5.65E+02 | 6.18E+00 | 6.76E+03 | 1.03E+02 | 2.60E-01 | 4.65E+02 | 1.26E+01 | 1.97E+02 | 1.41E+00 | 4.16E+02 | 1.05E+00 | 5.86E+01 | 5.81E+00 | 5.72E+00 |
| **Nadine M1** | 9.25E+01 | | 1.00E-01 | 9.45E+00 | 5.00E-02 | 1.39E+00 | 0.00E+00 | 1.01E+00 | 3.24E+03 | 0.00E+00 | 8.20E+00 | 2.20E+01 | 2.00E-02 | 1.69E+05 | 0.00E+00 | 2.01E+00 | 8.61E+00 | 5.26E+00 | 3.31E+03 | 5.13E+02 | 5.44E+00 | 6.22E+03 | 9.01E+01 | 2.80E-01 | 4.13E+02 | 1.22E+01 | 1.95E+02 | 1.63E+00 | 4.15E+02 | 1.17E+00 | 5.90E+01 | 5.32E+00 | 5.35E+00 |
| **Nadine C1** | 9.25E+01 | | 4.00E-02 | 9.64E+00 | 3.00E-02 | 3.50E-01 | 0.00E+00 | 3.50E-01 | 2.24E+03 | 0.00E+00 | 1.00E+01 | 2.06E+01 | 0.00E+00 | 1.71E+05 | 0.00E+00 | 1.65E+00 | 1.01E+01 | 4.54E+00 | 2.15E+03 | 4.65E+02 | 4.57E+00 | 5.90E+03 | 6.93E+01 | 2.40E-01 | 3.93E+02 | 8.78E+00 | 1.93E+02 | 1.78E+00 | 4.39E+02 | 1.21E+00 | 5.03E+01 | 4.77E+00 | 4.12E+00 |
| **Osama B2** | 1.24E+02 | | 1.00E-01 | 9.71E+00 | 2.00E-02 | 1.70E+00 | 5.80E-01 | 1.12E+00 | 1.72E+03 | 1.60E-01 | 9.08E+00 | 1.72E+01 | 0.00E+00 | 1.44E+05 | 0.00E+00 | 1.39E+00 | 4.20E+00 | 4.64E+00 | 1.93E+03 | 4.31E+02 | 3.71E+00 | 5.87E+03 | 4.82E+01 | 4.00E-01 | 3.79E+02 | 7.58E+00 | 1.83E+02 | 1.82E+00 | 4.13E+02 | 1.21E+00 | 2.71E+01 | 3.57E+00 | 3.44E+00 |
| **Osama C1** | 1.24E+02 | | 8.00E-02 | 9.59E+00 | 0.00E+00 | 8.60E-01 | 0.00E+00 | 8.60E-01 | 1.80E+03 | 0.00E+00 | 7.88E+00 | 1.80E+01 | 0.00E+00 | 1.37E+05 | 0.00E+00 | 1.37E+00 | 4.02E+00 | 4.09E+00 | 1.85E+03 | 4.14E+02 | 3.81E+00 | 5.76E+03 | 5.02E+01 | 2.20E-01 | 3.66E+02 | 7.27E+00 | 1.90E+02 | 1.42E+00 | 3.91E+02 | 1.18E+00 | 3.00E+01 | 3.34E+00 | 2.26E+00 |
| **Osama C2** | 1.24E+02 | | 1.40E-01 | 9.57E+00 | 4.00E-02 | 9.50E-01 | 0.00E+00 | 9.50E-01 | 3.22E+03 | 4.50E-01 | 1.06E+01 | 2.81E+01 | 2.00E-02 | 1.88E+05 | 0.00E+00 | 2.34E+00 | 1.80E+01 | 5.84E+00 | 3.45E+03 | 5.36E+02 | 5.76E+00 | 8.25E+03 | 1.06E+02 | 3.40E-01 | 4.65E+02 | 1.22E+01 | 2.49E+02 | 2.00E+00 | 4.78E+02 | 1.13E+00 | 8.82E+01 | 6.82E+00 | 6.66E+00 |
| **Osama M2** | 1.24E+02 | | 1.00E-01 | 9.58E+00 | 4.00E-02 | 1.05E+00 | 0.00E+00 | 1.05E+00 | 2.96E+03 | 4.00E-01 | 1.02E+01 | 2.40E+01 | 1.00E-02 | 1.94E+05 | 0.00E+00 | 2.18E+00 | 1.43E+01 | 6.95E+00 | 3.25E+03 | 5.12E+02 | 5.54E+00 | 8.02E+03 | 9.37E+01 | 3.70E-01 | 4.86E+02 | 1.12E+01 | 1.98E+02 | 2.07E+00 | 4.92E+02 | 1.36E+00 | 7.38E+01 | 6.41E+00 | 5.41E+00 |
| **Paul M** | 2.26E+01 | | 1.00E-01 | 9.41E+00 | 1.00E-01 | 2.24E+00 | 3.70E-01 | 1.87E+00 | 3.91E+03 | 9.10E-01 | 1.20E+01 | 2.21E+01 | 3.00E-02 | 2.59E+05 | 0.00E+00 | 2.88E+00 | 3.46E+01 | 6.16E+00 | 4.31E+03 | 5.03E+02 | 6.49E+00 | 7.67E+03 | 1.30E+02 | 5.10E-01 | 6.10E+02 | 1.35E+01 | 2.93E+02 | 2.04E+00 | 5.96E+02 | 1.17E+00 | 1.39E+02 | 9.85E+00 | 7.33E+00 |
| **Perez M** | 1.13E+03 | | 5.70E-01 | 8.54E+00 | 3.00E-02 | 1.41E+00 | 6.40E-01 | 7.70E-01 | 2.54E+03 | 1.60E-01 | 1.42E+01 | 2.38E+01 | 0.00E+00 | 1.18E+05 | 0.00E+00 | 1.75E+00 | 7.56E+00 | 4.87E+00 | 2.67E+03 | 4.60E+02 | 5.23E+00 | 7.33E+03 | 7.04E+01 | 3.90E-01 | 5.35E+02 | 9.85E+00 | 1.55E+02 | 1.24E+00 | 5.90E+03 | 1.08E+00 | 7.97E+01 | 5.48E+00 | 3.72E+00 |
| **Rana C1** | 2.19E+02 | | 1.20E-01 | 9.54E+00 | 3.00E-02 | 1.16E+00 | 0.00E+00 | 1.16E+00 | 2.28E+03 | 0.00E+00 | 7.56E+00 | 2.14E+01 | 0.00E+00 | 1.35E+05 | 0.00E+00 | 1.65E+00 | 4.51E+00 | 4.90E+00 | 2.29E+03 | 4.95E+02 | 4.61E+00 | 5.26E+03 | 6.45E+01 | 2.40E-01 | 3.59E+02 | 9.36E+00 | 2.51E+02 | 1.76E+00 | 3.64E+02 | 1.26E+00 | 3.82E+01 | 4.03E+00 | 4.21E+00 |
| **Rana C2** | 2.19E+02 | | 1.20E-01 | 9.33E+00 | 5.00E-02 | 1.39E+00 | 0.00E+00 | 1.39E+00 | 3.33E+03 | 0.00E+00 | 7.60E+00 | 2.50E+01 | 2.00E-02 | 1.70E+05 | 0.00E+00 | 2.30E+00 | 8.62E+00 | 5.56E+00 | 3.32E+03 | 5.63E+02 | 6.17E+00 | 6.72E+03 | 1.00E+02 | 2.70E-01 | 4.43E+02 | 1.25E+01 | 1.98E+02 | 1.50E+00 | 4.16E+02 | 1.14E+00 | 6.25E+01 | 5.49E+00 | 5.62E+00 |
| **Sara C1** | 1.92E+02 | | 4.00E-02 | 9.64E+00 | 3.00E-02 | 3.50E-01 | 0.00E+00 | 3.50E-01 | 2.23E+03 | 0.00E+00 | 1.00E+01 | 2.06E+01 | 0.00E+00 | 1.71E+05 | 0.00E+00 | 1.65E+00 | 1.01E+01 | 4.54E+00 | 2.15E+03 | 4.65E+02 | 4.57E+00 | 5.90E+03 | 6.93E+01 | 2.30E-01 | 3.95E+02 | 8.80E+00 | 1.94E+02 | 1.78E+00 | 4.40E+02 | 1.21E+00 | 5.02E+01 | 4.77E+00 | 3.89E+00 |
| **Sara C2** | 1.92E+02 | | 2.00E-02 | 9.59E+00 | 4.00E-02 | 4.20E-01 | 0.00E+00 | 4.20E-01 | 2.47E+03 | 0.00E+00 | 1.07E+01 | 3.24E+01 | 0.00E+00 | 1.75E+05 | 0.00E+00 | 1.87E+00 | 1.05E+01 | 4.65E+00 | 2.49E+03 | 4.96E+02 | 4.99E+00 | 6.09E+03 | 7.43E+01 | 2.60E-01 | 3.98E+02 | 9.67E+00 | 2.04E+02 | 5.64E+00 | 4.58E+02 | 1.14E+00 | 5.65E+01 | 5.45E+00 | 4.49E+00 |
| **Sara M** | 1.92E+02 | | 8.00E-02 | 9.56E+00 | 7.00E-02 | 9.20E-01 | 2.20E-01 | 7.00E-01 | 1.90E+03 | 0.00E+00 | 9.37E+00 | 2.25E+01 | 0.00E+00 | 1.77E+05 | 0.00E+00 | 1.50E+00 | 7.00E+00 | 4.65E+00 | 1.78E+03 | 4.49E+02 | 4.25E+00 | 5.64E+03 | 5.51E+01 | 2.20E-01 | 4.11E+02 | 7.92E+00 | 1.90E+02 | 3.65E+00 | 4.70E+02 | 8.70E-01 | 3.21E+01 | 3.71E+00 | 2.82E+00 |
| **Singing M** | 6.00E+02 | | 6.00E-02 | 9.32E+00 | 3.00E-02 | 2.58E+00 | 1.94E+00 | 6.40E-01 | 1.67E+03 | 0.00E+00 | 1.15E+01 | 1.66E+01 | 0.00E+00 | 1.05E+05 | 0.00E+00 | 1.29E+00 | 6.13E+00 | 4.36E+00 | 1.97E+03 | 3.90E+02 | 3.46E+00 | 5.74E+03 | 4.32E+01 | 3.10E-01 | 4.38E+02 | 7.05E+00 | 1.48E+02 | 1.08E+00 | 3.68E+02 | 1.21E+00 | 3.68E+01 | 4.06E+00 | 2.82E+00 |
