# Supplementary material for: Microbial Characterization of Qatari Barchan Sand Dunes
Source: PLoS One. 2016 Sep 21;11(9):e0161836. doi: 10.1371/journal.pone.0161836 (PMC5031452; doi:10.1371/journal.pone.0161836)
Supplement: S3 Table — (DOCX) [file pone.0161836.s007.docx]

**S3 Table**. Number of colony forming units (± Standard Error) in a Qatari Barchan dune using different quantification methods

| **Method** | **Counts** | **Culturability** |
| --- | --- | --- |
| Single Grain Plate Count | 6.8 ± 1.0 x 10^3^ colony forming grains /g | 1.20% |
| Filtered Extracts Plate Count | 2.0 ± 0.5 x 10^3^ CFUs/ g | 0.4% (grains)  3.8% (filters) |
| Direct Counts: Filter fluorescence | 5.3 ± 0.7 x 10^4^ cells/ g | NA |
| Direct Counts: Grain fluorescence | 5.3 ± 0.4 x 10^5^ cells/ g | NA |
