# Supplementary material for: Microbial Characterization of Qatari Barchan Sand Dunes
Source: PLoS One. 2016 Sep 21;11(9):e0161836. doi: 10.1371/journal.pone.0161836 (PMC5031452; doi:10.1371/journal.pone.0161836)
Supplement: S4 Table — (DOCX) [file pone.0161836.s008.docx]

**S4 Table.** Identity and accession number of the closest BLAST match to the 16S rRNA gene sequences of isolates recovered from the single grain cultivations and serial dilutions.

| **Closest BLAST Match** | **Representative Accession Number** | **No. of Isolates** | **% of Total** | **% Max Identity Range** |
| --- | --- | --- | --- | --- |
| ***Actinobacteria*** |  |  |  |  |
| *Arthrobacter* sp. H23 | KC442327 | 1 | 1.56% | 100 |
| *Arthrobacte*r sp. SaPRH3 | JQ806473 | 1 | 1.56% | 99 |
| *Dermacoccus* sp. F218T | KM188051 | 1 | 1.56% | 98 |
| *Dietzia* sp. 0705K4-1 | HM222663 | 1 | 1.56% | 100 |
| *Friedmanniella sagamiharensis* | AB445456 | 2 | 3.13% | 92-93 |
| *Kocuria* sp. AD-G11 | HQ690914 | 3 | 4.69% | 97-99 |
| *Kocuria* sp. M-10 | JX502618 | 2 | 3.13% | 99-100 |
| *Kocuria* sp. M68 | JX502620 | 1 | 1.56% | 99 |
| *Micrococcus* sp. TA1 | AB524880 | 1 | 1.56% | 98 |
| *Micromonospora* sp. CS1-12 | AB981048 | 1 | 1.56% | 94 |
| *Streptomyces heliomycini* strain 173574 | EU593729 | 1 | 1.56% | 100 |
| *Streptomyces humidus* strain cfcc3146 | FJ883751 | 2 | 3.13% | 99-100 |
| *Streptomyces lavendulocolor* strain 173390 | EU570688 | 2 | 3.13% | 98 |
| *Streptomyces* sp. DRL63 | FJ911542 | 5 | 7.81% | 97-100 |
| *Streptomyces* sp. MBRC-34 | KC179795 | 8 | 12.50% | 99 |
| *Streptomyces* sp. MS-1 | JN578482 | 5 | 7.81% | 98-100 |
|  | **Total** | **37** | **58%** |  |
| ***Firmicutes*** |  |  |  |  |
| *Bacillus firmu*s strain DHXJ20 | JN244990 | 1 | 1.56% | 99 |
| *Bacillus pumilus* strain GR32 | KC771047 | 1 | 1.56% | 99 |
| *Bacillus pumilus* strain HT-Z75-B1 | KJ526888 | 2 | 3.13% | 99-100 |
| *Bacillus simplex* strain ArzA-13A | JQ929012 | 1 | 1.56% | 99 |
| *Bacillus* sp. 4-20 | AJ781707 | 7 | 10.94% | 99-100 |
| *Bacillus* sp. CMJ1-5 | KC119114 | 1 | 1.56% | 99 |
| *Bacillus subtilis* strain CM 2 | KC920909 | 1 | 1.56% | 99 |
| *Paenibacillus lautus* strain SK21 | FJ974058 | 1 | 1.56% | 99 |
| *Paenibacillus* sp. DYJL13 | HQ317156 | 2 | 3.13% | 99 |
|  | **Total** | **17** | **27%** |  |
| ***Proteobacteria*** |  |  |  |  |
| *Phenylobacterium* sp. W2.09-62 | JX458456 | 1 | 1.56% | 96 |
| *Pseudomonas putida* strain QAU67 | KC679991 | 1 | 1.56% | 99 |
| *Pseudomonas* sp. LM5 | EU821341 | 1 | 1.56% | 98 |
| *Pseudomonas* sp. SI-2 | AY729996 | 3 | 4.69% | 99 |
| P*seudomonas stutzeri* | HF952671 | 2 | 3.13% | 98-99 |
| *Altererythrobacter dongtanensis* strain JM27 | NR_108695 | 2 | 3.13% | 96-97 |
|  | **Total** | **10** | **15%** |  |
