# Supplementary material for: Microbial Characterization of Qatari Barchan Sand Dunes
Source: PLoS One. 2016 Sep 21;11(9):e0161836. doi: 10.1371/journal.pone.0161836 (PMC5031452; doi:10.1371/journal.pone.0161836)
Supplement: S5 Table — A. Results of multiple linear regression of significant environmental variables (p<0.05) to alpha diversity and Gammaproteobacteria relative abundance. B. Students Ttest demonstrated the mean Gammaproteobacterial abundance was greater for dunes located downwind of a camel farm (+), than for dunes that were not (-) (P = 0.012). (DOCX) [file pone.0161836.s009.docx]

**S5 Table. A**. Results of multiple linear regression of significant environmental variables (p<0.05) to alpha diversity and Gammaproteobacteria relative abundance. **B**. Students Ttest demonstrated the mean Gammaproteobacterial abundance was greater for dunes located downwind of a camel farm (+), than for dunes that were not (-) (P=0.012).

|  | Alpha Diversity | | | Gammaproteobacteria Relative Abundance | | |
| --- | --- | --- | --- | --- | --- | --- |
| Modeled Variables | β (Std Error) | t-value | p-value | β (Std Error) | t-value | p-value |
| Dune Size | -0.002 (0.000) | -3.466 | 0.001 | 0.001 (0.000) | 2.447 | 0.018 |
| Nitrate | 0.261 (0.092) | 2.848 | 0.014 | -0.077 (0.033) | -2.301 | 0.037 |
| pH | -1.137 (0.309) | -3.675 | 0.003 | 0.515 (0.109) | 4.723 | 0.000 |
| Aluminum | -0.001 (0.001) | -2.446 | 0.029 | 0.001 (0.000) | 5.346 | 0.000 |
| Arsenic | -0.965 (0.153) | -6.294 | 0.000 | 0.249 (0.056) | 4.417 | 0.001 |
| Barium | 0.236 (0.032) | 7.364 | 0.000 | -0.071 (0.010) | -7.048 | 0.000 |
| Iron | 0.001 (0.001) | 8.514 | 0.000 | 0.000 (0.000) | -8.635 | 0.000 |
| Potassium | 0.004 (0.002) | 3.916 | 0.002 | -0.001 (0.000) | -4.089 | 0.001 |
| Lithium | -0.996 (0.308) | 2.404 | 0.032 | -0.001 (0.001) | -2.520 | 0.024 |
| Magnesium | -0.001 (0.001) | -3.237 | 0.006 | 0.000 (0.000) | 8.600 | 0.000 |
| Phosphorous | -0.009 (0.003) | -7.157 | 0.000 | 0.004 (0.001) | 4.462 | 0.001 |
| Sulfur | 0.001 (0.000) | -3.524 | 0.004 | 0.000 (0.000) | -2.651 | 0.019 |
| Titanium | 0.019 (0.004) | 6.431 | 0.000 | -0.006 (0.002) | -4.005 | 0.001 |

A.

| Camel Farm + | | Camel Farm - | |
| --- | --- | --- | --- |
| Dune | % Gamma | Dune | %Gamma |
| Qatar | 69 | Paul | 48 |
| Rana | 72 | Sara | 19 |
| Camera | 69 | Brook | 57 |
| Baya | 74 | Osama | 91 |
| Michel | 81 | Dana | 36 |
| Chris | 94 | Nadine | 28 |
| Average | 76.5 |  | 46.5 |

B.
