# Supplementary material for: Microbial Characterization of Qatari Barchan Sand Dunes
Source: PLoS One. 2016 Sep 21;11(9):e0161836. doi: 10.1371/journal.pone.0161836 (PMC5031452; doi:10.1371/journal.pone.0161836)
Supplement: S6 Table — (DOCX) [file pone.0161836.s010.docx]

| **MG-RAST Details** | **Michel** | **Nadine** |
| --- | --- | --- |
| Post QC: bp Count | 109,134,600 bp | 39,157,200 bp |
| Post QC: Sequences Count | 727,564 | 261,048 |
| Post QC: Mean Sequence Length | 150 ± 0 bp | 150 ± 0 bp |
| Post QC: Mean GC percent | 46 ± 12 % | 48 ± 15 % |
| Processed: Predicted Protein Features | 533,101 | 219,779 |
| Processed: Predicted rRNA Features | 110,034 | 78,323 |
| Alignment: Identified Protein Features | 179,885 | 70,102 |
| Alignment: Identified rRNA Features | 2,595 | 228 |
| Annotation: Identified Functional Categories | 155,946 | 61,062 |

**S6 Table.** MG-RAST metagenomic sequencing details.
