# Supplementary material for: Microbial Characterization of Qatari Barchan Sand Dunes
Source: PLoS One. 2016 Sep 21;11(9):e0161836. doi: 10.1371/journal.pone.0161836 (PMC5031452; doi:10.1371/journal.pone.0161836)
Supplement: S7 Table — (DOCX) [file pone.0161836.s011.docx]

|  | **Nadine** | | **Michel** | |
| --- | --- | --- | --- | --- |
| **BACTERIA** |  |  |  |  |
|  | **% Metagenome** | **% Bacteria** | **% Metagenome** | **% Bacteria** |
|  |  |  |  |  |
| Archaea | **2.10%** | **N/A** | **2.18%** | **N/A** |
| Bacteria | **93.46%** | **N/A** | **87.53%** | **N/A** |
| Eukarya | **4.15%** | **N/A** | **9.54%** | **N/A** |
| Viruses | **0.09%** | **N/A** | **0.37%** | **N/A** |
| Other sequences | **0.00%** | **N/A** | **0.03%** | **N/A** |
| Unassigned | **0.15%** | **N/A** | **0.29%** | **N/A** |
| Unclassified sequences | **0.05%** | **N/A** | **0.05%** | **N/A** |
| Total | **100.0%** | **N/A** | **100.0%** | **N/A** |
| ***Most abundant bacterial phyla for either dune*** | | | | |
| *Actinobacteria* | **41.97%** | **44.90%** | **14.40%** | **16.45%** |
| *Firmicutes* | **23.18%** | **24.80%** | **23.38%** | **26.71%** |
| *Proteobacteria* | **12.13%** | **12.98%** | **21.33%** | **24.37%** |
| *Bacteroidetes* | **9.48%** | **10.14%** | **19.68%** | **22.48%** |
| *Chloroflexi* | **1.67%** | **1.79%** | **1.21%** | **1.38%** |
| *Cyanobacteria* | **1.13%** | **1.21%** | **2.78%** | **3.17%** |
| Total (# of phyla) | **89.56% (27)** | **95.83%** | **82.769% (27)** | **94.56%** |
| ***Most abundant bacterial classes for either dune*** | | | | |
| *Actinobacteria (*class*)* | **41.97%** | **44.90%** | **14.40%** | **16.45%** |
| *Bacilli* | **20.35%** | **21.77%** | **19.09%** | **21.81%** |
| *Alphaproteobacteria* | **3.97%** | **4.25%** | **4.00%** | **4.57%** |
| *Cytophagia* | **3.54%** | **3.79%** | **5.02%** | **5.74%** |
| *Gammaproteobacteria* | **3.49%** | **3.73%** | **10.30%** | **11.76%** |
| *Flavobacteriia* | **2.55%** | **2.73%** | **5.27%** | **6.02%** |
| *Bacteroidia* | **1.40%** | **1.49%** | **5.24%** | **5.99%** |
| Total (# of classes) | **77.26% (54)** | **82.67%** | **63.32% (55)** | **72.34%** |
| ***Most abundant bacterial families for either dune*** | | | | |
| Bacillaceae | **17.20%** | **18.40%** | **13.06%** | **14.92%** |
| *Streptomycetaceae* | **9.27%** | **9.92%** | **4.67%** | **5.34%** |
| *Pseudonocardiaceae* | **5.19%** | **5.55%** | **1.26%** | **1.44%** |
| *Micromonosporaceae* | **3.69%** | **3.95%** | **0.56%** | **0.64%** |
| *Nocardioidaceae* | **3.23%** | **3.46%** | **0.67%** | **0.76%** |
| *Enterobacteriaceae* | **0.59%** | **0.63%** | **5.32%** | **6.08%** |
| *Flavobacteriaceae* | **2.33%** | **2.49%** | **4.78%** | **5.46%** |
| *Cytophagaceae* | **2.73%** | **2.92%** | **4.11%** | **4.69%** |
| Total (# of phyla) | **27.03% (242)** | **28.93%** | **21.37% (257)** | **24.41%** |
| ***Most abundant bacterial genera for either dune*** | | | | |
| *Bacillus* | **12.73%** | **13.63%** | **9.88%** | **11.28%** |
| *Streptomyces* | **9.16%** | **9.80%** | **4.63%** | **5.28%** |
| *Geobacillus* | **3.04%** | **3.25%** | **2.08%** | **2.38%** |
| *Mycobacterium* | **3.02%** | **3.24%** | **1.71%** | **1.95%** |
| *Salinispora* | **2.42%** | **2.59%** | **0.32%** | **0.37%** |
| *Bacteroides* | **0.82%** | **0.87%** | **3.79%** | **4.32%** |
| *Escherichia* | **0.13%** | **0.14%** | **2.86%** | **3.27%** |
| Total (# of genera) | **31.32% (576)** | **33.51%** | **25.26% (664)** | **28.86%** |
| ***Most abundant gammaproteobacterial genera of either dune*** | | | | |
| *Pseudomonas* | **0.43%** | **0.46%** | **0.66%** | **0.75%** |
| *Acinetobacter* | **0.19%** | **0.20%** | **1.00%** | **1.14%** |
| *Xanthomonas* | **0.19%** | **0.20%** | **0.24%** | **0.28%** |
| *Shewanella* | **0.17%** | **0.18%** | **0.34%** | **0.39%** |
| *Vibrio* | **0.14%** | **0.15%** | **0.29%** | **0.33%** |
| *Escherichia* | **0.13%** | **0.14%** | **2.86%** | **3.27%** |
| *Shigella* | **0.03%** | **0.03%** | **0.52%** | **0.59%** |
| *Salmonella* | **0.05%** | **0.06%** | **0.42%** | **0.48%** |
| Total (# of genera) | **0.90% (86)** | **0.97%** | **5.68% (102)** | **6.49%** |
|  | **Nadine** | | **Michel** | |
| **EUKARYA** |  |  |  |  |
|  | **% Metagenome** | **% Eukaryota** | **% Metagenome** | **% Eukaryota** |
| ***Most abundant eukaryotic phyla for either dune*** | | | | |
| *Chordata* | **1.55%** | **37.34%** | **6.42%** | **67.31%** |
| *Arthropoda* | **1.42%** | **34.19%** | **0.54%** | **5.65%** |
| *Ascomycota* | **0.33%** | **7.88%** | **0.63%** | **6.62%** |
| *Apicomplexa* | **0.11%** | **2.58%** | **0.52%** | **5.40%** |
| *Nematoda* | **0.08%** | **1.81%** | **0.07%** | **0.08%** |
| *Streptophyta* | **0.00%** | **0.00%** | **0.67%** | **7.03%** |
| Total (# of phyla) | **3.478% (14)** | **83.81%** | **8.85% (20)** | **92.09%** |

**S7 Table.** MG-RAST metagenomic organism abundance analysis for Nadine and Michel dunes, based on Representative Hit Classification of the M5NR database.
